# Risk identification and prediction of complaints and misconduct against health practitioners: a scoping review

**DOI:** 10.1093/intqhc/mzad114

**Published:** 2023-12-29

**Authors:** Yufeng Wang, Sanyogita (Sanya) Ram, Shane Scahill

**Affiliations:** School of Pharmacy, Faculty of Medical and Health Sciences, University of Auckland, Level 3, Building 503, 85 Park Road, Grafton, Auckland 1023, New Zealand; School of Pharmacy, Faculty of Medical and Health Sciences, University of Auckland, Level 3, Building 503, 85 Park Road, Grafton, Auckland 1023, New Zealand; School of Pharmacy, Faculty of Medical and Health Sciences, University of Auckland, Level 3, Building 503, 85 Park Road, Grafton, Auckland 1023, New Zealand

**Keywords:** risk identification, risk prediction, complaints, misconduct, health practitioners

## Abstract

Identifying the risk and predicting complaints and misconduct against health practitioners are essential for healthcare regulators to implement early interventions and develop long-term prevention strategies to improve professional practice and enhance patient safety. This scoping review aims to map out existing literature on the risk identification and prediction of complaints and misconduct against health practitioners. This scoping review followed Arksey and O’Malley’s five-stage methodological framework. A comprehensive literature search was conducted on MEDLINE, EMBASE, and CINAHL databases and finished on the same day (6 September 2021). Articles meeting the eligibility criteria were charted and descriptively analysed through a narrative analysis method. The initial search generated 5473 articles. After the identification, screening, and inclusion process, 81 eligible studies were included for data charting. Three key themes were reported: methods used for identifying risk factors and predictors of the complaints and misconduct, synthesis of identified risk factors and predictors in eligible studies, and predictive tools developed for complaints and misconduct against health practitioners. The findings reveal that risk identification and prediction of complaints and misconduct are complex issues influenced by multiple factors, exhibiting non-linear patterns and being context specific. Further efforts are needed to understand the characteristics and interactions of risk factors, develop systematic risk prediction tools, and facilitate the application in the regulatory environment.

## Introduction

In recent years, health regulators have shown an increasing inclination towards proactive and data-driven approaches to oversight [1]. Complaints and misconduct are recognized as valuable resources for understanding patient dissatisfaction and harm, as well as detecting problematic practitioner practices [2, 3]. Regulatory agencies worldwide consider complaints and misconduct as crucial components of their risk management frameworks. For instance, in the USA, the Vanderbilt Center for Patient and Professional Advocacy (CPPA) has developed a Patient Advocacy Reporting System (PARS) complaint programme in collaboration with hospitals and medical groups to identify physicians at high risk of complaints effectively [4]. Similarly, national schemes have been implemented in Australia and New Zealand to regulate health professions, with a particular focus on complaints and disciplinary data [5]. These developments were noted as offering ‘opportunities for researchers, policymakers and regulators to move towards evidence-based regulation’ [6].

In related fields, a growing number of studies have explored complaints and misconduct against health practitioners. Numerous investigations have revealed that the occurrence of such complaints and misconduct is not entirely random, with a small proportion of practitioners accounting for up to 50% of claims [7–9]. Certain research has identified specific characteristics associated with high-risk health practitioners, including demographics such as gender, age, and speciality [10, 11]. Moreover, beyond these “personal” or “human” factors, practice or system characteristics, such as workload [12] and practice setting [11], have emerged as discernible indicators of medico-legal matters. Despite the growing body of research in this field, there is a lack of reviews that comprehensively examine the risk of complaints and misconduct against health practitioners, especially when viewed through a predictive and prospective lens.

This study adopts a broad perspective with the goal of providing a comprehensive understanding of existing literature on the risk and prediction of complaints and misconduct against health practitioners. To achieve this, a broad scoping review methodology was employed due to its advantages in mapping a wide range of available evidence, identifying key characteristics or factors related to a concept, and conveying the breadth and depth of a field [13–15]. This study seeks to address the following three specific questions: (i) what methods have been used to identify risk factors and predictors of complaints and misconduct against health practitioners? (ii) What are the patterns and taxonomy of risk factors and predictors of complaints and misconduct against health practitioners? (iii) What predictive models or tools have been developed to identify risks for complaints or misconduct against health practitioners prospectively?

## Methods

### Study design

This study utilized the well-established five-stage methodological framework proposed by Arksey and O’Malley [13], which is widely recognized and commonly used in scoping reviews. The five stages include (i) identifying the research question, (ii) identifying relevant studies, (iii) study selection, (iv) charting the data, and (v) collating, summarizing, and reporting the results. To ensure rationality and integrity, the Preferred Reporting Items for Systematic Reviews and Meta-Analyses (PRISMA) extension for Scoping Reviews (PRISMA-ScR) checklist was also applied to report the findings [16].

### Research question

The research question was formulated by following the Population, Concept, and Context framework [17]. In this study, the Population encompasses all health practitioners. The Concept focuses on the identification and prediction of risks associated with complaints and misconduct. Within the scope of this study, the term “complaints and/or misconduct” is a broad term that encompasses various related concepts, including complaints, misconducts, disciplinary matters, malpractices, grievances, misbehaviours, medico-legal cases, medical errors, disputes, criminal cases, and claims that are against health practitioners. There are no geographic or contextual restrictions. Consequently, the following research question was posited: what is known from the existing literature about the identification and prediction of risks associated with complaints and misconduct against health practitioners?

### Search strategy

The search strategy was developed over several research team meetings based on three sets of primary concepts: risk identification or prediction, health practitioners, and complaints and/or misconduct. A search of literature was conducted on 6 September 2021, using three databases: Medline via PubMed (1946 to the present), EMBASE via Ovid (1980 to the present), and CINHAL Plus via EBSCOhost (1937 to the present). All searches were subject to date and language limitations, with only English articles published since 2000 being included. Although the search was pre-planned in advance, no formal protocol was registered for this review. A full search strategy for each database is available in the supplementary file (Supplementary Appendix A).

### Screening and selection process

The screening and selection process was conducted in three stages. Firstly, the search results were imported into Endnote software. Duplicates were removed using the software’s built-in function, followed by further manual checking. Subsequently, the titles and abstracts of all imported studies were screened based on the inclusion criteria outlined in Table 1. Additionally, the reference lists of review papers were searched for additional relevant publications (i.e. backward snowballing) [13, 18], ensuring comprehensive coverage of evidence. Finally, exclusion criteria were developed for full-text screening, and the final decision regarding the inclusion of the remaining articles was made based on the refined eligibility criteria.

**Table 1.** Inclusion and exclusion criteria.

Inclusion criteria	Exclusion criteria
1. Original research articles describing experimental or observational investigations or review articles using formal methods to explore the identification or prediction of the risk of complaints and misconduct against health practitioners. 2. The primary focus of the study was on complaints, misconducts, disciplinary matters, malpractices, grievances, misbehaviours, medico-legal cases, medical errors, disputes, criminal cases, and claims against health practitioners (collectively referred to as “complaints and/or misconduct”). 3. Published in English since 2000.	1. Studies that focused on: (i) health complaints that referred to patients’ bodily disorders, diseases, symptoms, or distress; (ii) defensive medicine. 2. The following types of prediction studies: (i) did not target health practitioners or only focused exclusively on veterinarians; (ii) focused solely on predicting specific outcomes of complaints or misconduct (such as payment or liability); (iii) only analysed patient or complainant-related risk factors. 3. Publications in the form of commentary, editorials, letters, notes, news, communications, books, case reports, theoretical essays, conference abstracts, and oral presentations. 4. Non-English studies or publications before 2000. 5. The completed text was not available through the university library service.

To expedite the process, a rapid review approach “single screening” was adopted for abstract and full-text screening [19]. This widely accepted rapid review approach offers a more efficient analysis and quicker generation of evidence portfolio. However, it does carry the risk of potentially missing relevant studies. Nevertheless, given the extensive number of studies identified through the searching and screening processes, any missing studies are expected to have minimal impact on the overall summarizing and dissemination of these research findings [20, 21].

Throughout the searching, screening, and assessing process, the research team held regular meetings to discuss the eligible criteria, key concepts, and observations. One reviewer initially assessed each article, with any uncertainties resolved through discussions with two advisors who had expertise in pharmacy, organization management, and law until a consensus was reached.

### Data extraction, analysis, and report

An Excel^®^ sheet was created to extract the following information: author(s), title, journal, year of publication, location, methodology, data source, year of the study conducted, involved professional(s), aims, key findings, and conclusions. A narrative synthesis analysis method recommended by Arksey and O’Malley (2005) was then performed, including numerical statistical summaries, textual commentaries, and tabular and graphical representations [22].

## Results

### Search results and characteristics

**Figure 1:**
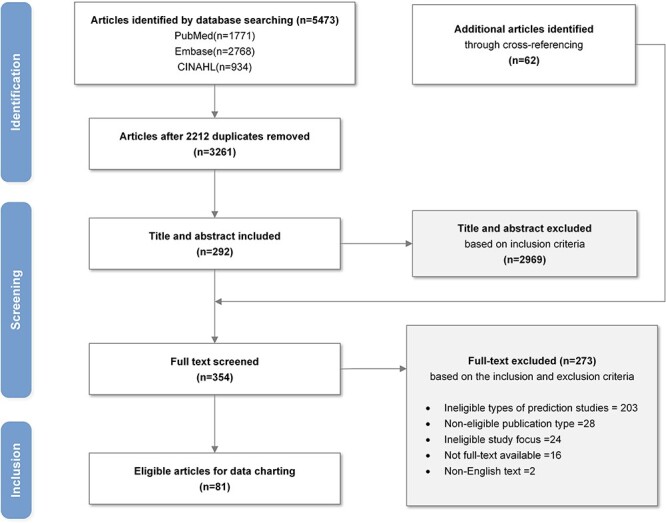
PRISMA flowchart for study selection.

The database search initially yielded 5473 articles. After removing 2212 duplicates, a total of 3261 articles were reviewed in title and abstract, and 292 were retained for full-text screening. Also included were 62 articles through screening the reference list of eight systematic reviews and traditional literature reviews. Through the full-text screening of 354 articles, 81 articles were finally included for data charting. The complete identification, screening, and inclusion process is illustrated in the PRISMA-ScR flowchart (Fig. 1). The supplementary file (Supplementary Appendix B) provides basic information on eligible studies.

As seen in Table 2, the number of identified articles has been increasing in recent years, with two peaks observed between 2011–2013 (*n* = 16) [7, 23–37] and 2018–2021(*n* = 27) [1, 2, 4, 9–12, 38–57]. Over three-quarters (77.78%) of these studies were conducted in the USA (*n* = 37, 45.68%) [4, 9, 24, 25, 29, 31, 36, 41, 43, 49, 52–55, 57–79], Australia (*n* = 14, 17.28%) [1, 2, 7, 8, 34, 37, 44, 45, 51, 56, 80–83], Canada (*n* = 6, 7.41%) [26, 28, 47, 84–86], and Denmark (*n* = 6, 7.41%) [27, 30, 32, 46, 48, 50]. Quantitative methods were predominantly used (73, 90.12%) [2, 4, 7–9, 12, 23–38, 41–85, 87–92], with no pure qualitative studies identified. The majority of studies (70, 86.42%) focused on physicians or doctors [2, 4, 7–12, 23, 25–27, 29, 30, 32, 34–41, 43, 44, 47–50, 52–79, 81–87, 89–94], while fewer studies targeted on pharmacists (*n* = 1) [33], nurses (*n* = 1) [31], and dentists (*n* = 3) [24, 42, 45]. Additionally, six studies considered broad or unspecified health practitioners [1, 28, 46, 51, 80, 88].

**Table 2.** Quantitative description of identified publications.

Category	*N*	%
**Publication year**		
2000	2	2.47
2002	2	2.47
2003	4	4.94
2004	3	3.70
2005	3	3.70
2006	2	2.47
2007	2	2.47
2008	2	2.47
2009	4	4.94
2010	2	2.47
2011	5	6.17
2012	5	6.17
2013	6	7.41
2014	4	4.94
2015	4	4.94
2016	2	2.47
2017	2	2.47
2018	8	9.88
2019	7	8.64
2020	7	8.64
2021(September)	5	6.17
**Locations from where the research data collected**		
USA	37	45.68
Australia	14	17.28
Canada	6	7.41
Denmark	6	7.41
Others^a^	5	6.17
UK	4	4.94
China	4	4.94
Italy	1	1.23
Norway	1	1.23
Japan	1	1.23
New Zealand	1	1.23
Greece	1	1.23
**Study design**		
Quantitative	73	90.12
Mixed method	3	3.70
Review	5	6.17
**Focused health professions**		
Physicians/doctors	70	86.42
Broad/unspecified health practitioners	6	7.41
Dentists	3	3.70
Nurses	1	1.23
Pharmacists	1	1.23
**Total**	81	100

a Review articles that collected data from more than one country.

### Methods used for identifying risk factors and predictors

The majority of the eligible studies (*n* = 76) adopt statistical analysis methods to identify risk factors or predictors of complaints and misconduct against health practitioners, encompassing 73 quantitative studies and 3 mixed-method studies. Specifically, 19 studies compared the characteristics and identified high-risk characteristics of complaints and misconduct cases by conducting significance tests such as the Chi-square test, Student’s *t*-test, and Mann–Whitney test. Additionally, 57 studies further examine the risk factors associated with complaints or misconduct through regression models, such as Poisson regression [47], logistical regression, recurrent-event survival analysis, and generalized estimating equation. In five other eligible review articles, three employed traditional narrative review analysis, with two utilizing meta-analysis methods for analysis for the identification of risk factors.

### Synthesis of identified risk factors and predictors

From the eligible 81 studies, three main categories of risk factors and predictors have been identified: health practitioners’ factors, system and environment factors, and complaint/misconduct issues. The synthesises and detailed findings for each eligible study are provided in Supplementary Appendix C and D.

**Figure 2:**
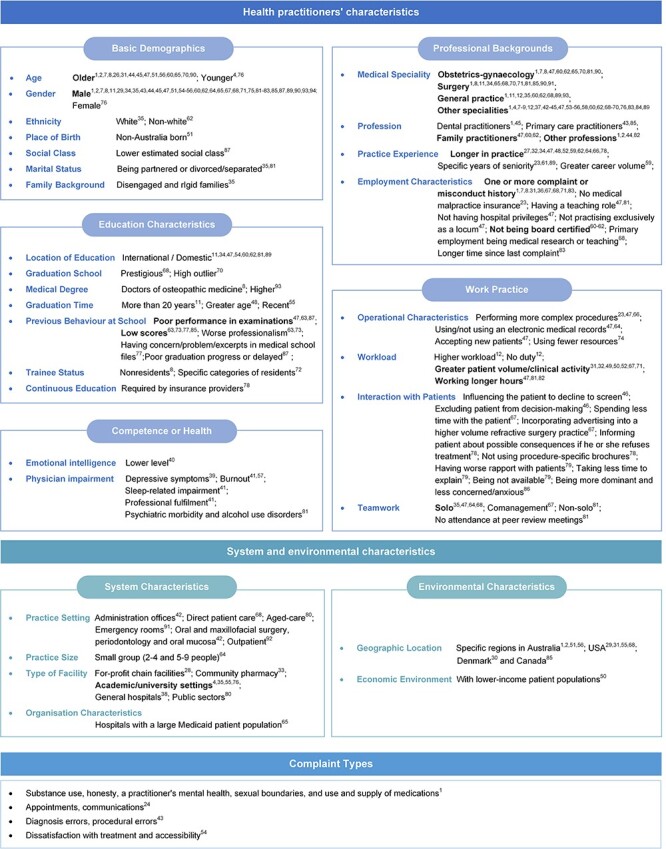
Risk factors for predicting complaints and misconduct against health practitioners.

Note: Risk factors in bold were identified in the literature more than three times.

Health practitioners’ factors were the most frequently mentioned, encompassing various aspects related to their basic demographics (age, gender, ethnicity, place of birth, social class, marital status, and family background), education characteristics (location of education, graduation school, medical degree, graduation time, previous behaviour at school, trainee status, and continuous education), competence or health (emotional intelligence and physician impairment), professional backgrounds (medical speciality, profession, practice experience, and employment characteristics), and work practice (operational characteristics, workload, interaction with patients, and teamwork). The specific risk factors and predictors extracted from eligible studies are presented in Fig. 2. However, it should be noted that only a limited number of factors were universally validated in more than three studies. These included older age [1, 2, 7, 8, 26, 31, 44, 45, 47, 51, 56, 60, 65, 70, 90], male gender [1, 2, 7, 8, 11, 29, 34, 35, 43–45, 47, 51, 54–56, 60, 62, 64, 65, 67, 68, 71, 75, 81–83, 85, 87, 89, 90, 93, 94], poor performance in examinations [47, 63, 73], low scores [63, 73, 77, 85], specific specialties (surgery [1, 8, 11, 34, 65, 68, 70, 71, 81, 85, 90, 91], obstetrics-gynaecology [1, 7, 8, 47, 60, 62, 65, 70, 81, 90], and general practice [1, 11, 12, 35, 60, 62, 68, 89, 93]), the professions of family practitioners [47, 60, 62], longer practice time [27, 32, 34, 47, 48, 52, 59, 62, 64, 66, 78], one or more previous claims history [1, 7, 8, 31, 36, 67, 71, 83, 95], not being board certified [60–62], greater patient volume/clinical activity [31, 32, 49, 50, 52, 67, 71], working longer hours [47, 81, 82], and solo practice [35, 47, 64, 68].

System and environment risk factors pertain to the factors related to the system, organization, or the broad environment in which the health practitioner practices. System factors included specific practice settings, practice size, facility type, and organization characteristics. One predominant system factor identified in the studies was the academic/university settings. Regarding environmental factors, several studies conducted in the USA [29, 31, 55, 68], Australia [1, 2, 51, 56], Denmark [30], and Canada [85] examined the relationship between geographic regions and the risk of complaints and misconduct. In addition, a Danish study found that General Practitioners serving lower-income patients were more likely to receive complaints or disciplinary board criticisms [50].

In terms of the issue of complaints and misconduct, four studies reported specific issues that pose a high risk. An Australian study found that complaints related to substance use, honesty, mental health, sexual boundaries, and medication use and supply were strongly recurrent [1]. In a dental school setting, appointments had a high complaint rate, with communication being the second-highest complaint type overall [24]. In the USA, one study revealed that plaintiffs overwhelmingly alleged diagnosis and procedural errors [43]. Another American study identified dissatisfaction with treatment and accessibility as the two most common complaint types among high-risk otolaryngologists [54].

### Predictive tools for complaints and misconduct analysis

Three practical predictive tools have been developed for regulating health practitioners (Table 3). The first tool is the CPPA PARS database developed in the USA [55]. This database generates a risk score and establishes a complaint-type profile for each physician through analysis of the complaint data, allowing institutions to identify physicians at higher risk [55, 58, 76]. The other tool is the Predicted Risk of New Event (PRONE) score, proposed and tested by Spittal *et al*. in Australia [1, 83]. It uses a 22-point scoring system consisting of four predictors: speciality, gender, previous complaint numbers, and the time since the last complaint. This scoring system helps to assess a doctor’s future likelihood of complaint [83]. In 2019, an updated Predicted Risk of New Event for Health Practitioners (PRONE-HP) version was released, incorporating two additional predictors: practice location and complaint issues [1].

**Table 3.** Predictive model and tools for complaints and misconduct analysis.

Predictive tools	Objective	Setting	Variables	Risk score measurement
CPPA PARS [55]	Physician	The USA	Complaint types	Each unique complaint embedded in a complaint report is assigned to one of the six complaint categories: care and treatment, communication, access and availability, concern for patient and family, safety of environment, and billing. The complaint-type profile for each physician reflects the distribution of complaints across these categories. The PARS system analyses these complaints and generates a risk score for each physician. The specific calculation method has not been disclosed.
PRONE score [83]	Doctor	Australia	A doctor’s specialty, sex, the number of previous complaints, and the time since the last complaint	Risk increased monotonically with PRONE score. Doctors with a score between 0 and 2 had a 2-year complaint risk of 14.2%. Doctors with PRONE scores between 15 and 17 had a subsequent complaint risk of 87.8%. Specialty: Anaesthesia (0), radiology (0), other specialties (0), internal medicine (1), ophthalmology (2), general practice (2), psychiatry (3), orthopaedic surgery (3), other surgery (3), general surgery (3), obstetrics and gynaecology(3), dermatology (4), and plastic surgery(6) Sex: Female (0) and male (2) Number of previous complaints: 1 (0), 2 (1), 3 (2), 4 (4), 5–6 (5), 7–8 (6), 9 (7), and 10+ (11) Time since the last complaint: 1–2 years (0), 6 months to 1 year (1), <6 months (2)
PRONE-HP score [1]	Health practitioners, including doctors, nurses and midwives, dental practitioners, psychologists, pharmacists, chiropractors, optometrists, osteopaths, physiotherapists, podiatrists, aboriginal and Torres-Strait Islander (ATSI) health practitioners, Chinese medicine practitioners, medical radiation practitioners, and occupational therapists	Australia	Sex, age, profession and specialty, practice location, number of prior complaints, and complaint issue	Practitioners with a score ≤4 had a 1% chance of a complaint within 24 months and those with a score ≥35 had a >85% chance. Sex: Female (0) and male (2) Age: ≤25 years (0), 26–35 years (0), 36–45 years (3), 46–55 years (4), 56–65 years (4), and 66–75 years (4) Profession/specialty: Doctor: general practice (14), surgery (16), obstetrics and gynaecology (17), physician (13), psychiatry (17), anaesthesia (10), radiology (11), emergency and intensive care unit (ICU) (10), non-clinical (9), non-specialist (12); nurse: registered nurse (4), enrolled nurse (4); midwife (0); dental: dentists and dental prosthetist (15), other dental practitioners (7); psychologist (11); pharmacist (10); ATSI practitioner (9); Chinese medicine practitioner (7); chiropractor (11); medical radiation practitioner (0); occupational therapist (2); optometrist (7); osteopath (6); physiotherapist (3); podiatrist (9) Practice location: Major cities of Australia (0), inner/outer regional Australia (1), and remote/very remote Australia (2) Number of prior complaints: 0 (0), 1 (6), 2 (8), 3 (10), 4–6 (12), ≥7 (15) Complaint issue: Health: physical health (3), mental health (7), substance use (9) Conduct: Records & reports (3), use or supply of medications (5), honesty (8), fees and servicing (3), interpersonal behaviour (3), sexual boundaries (5), compliance with conditions (2), and other conduct issues (4) Performance: Prescribing or dispensing (3), procedures (3), and treatment, communication, and other clinical issues (2)

## Discussion

### Statement of principal findings

The principal findings of this scoping review provide a comprehensive overview of the existing literature on risk identification and prediction related to complaints and misconduct against health practitioners. Although the included studies demonstrate a broad geographic and professional diversity, 60% were conducted in the USA and Australia, and 86% primarily focused on physicians. This observation suggests a need for more diverse and inclusive research encompassing a wider range of health practitioners in different contexts.

The identified risk factors and predictors fall into three categories: health practitioners’ factors, system and environment factors, and issues of complaints/misconduct. Health practitioners’ characteristics were the most frequently reported risk factors. Our analysis consistently found certain characteristics recurring as risk factors in more than three studies, such as older age, male gender, poor performance at school, complaints or misconduct history, longer in practice, and higher workloads. However, when investigating specific risk factors, it becomes evident that these factors are context specific and profession dependent. For example, studies vary in findings on age’s impact on complaints and misconduct against health practitioners [1, 4, 76]. Furthermore, different analytical perspectives reveal diverse patterns. Physician-level analysis shows higher operative volume heightens liability risk due to potential adverse events in every patient interaction, while institutionally, greater clinical volume correlates with lower mortality rates for some surgeries [49]. This highlights the need for targeted and comprehensive studies to enhance the specific field across different settings and professions.

It is worth noting that environment and organization characteristics play an important role in risk assessment and prediction. While our review has included isolated findings mentioning these factors as potential risk factors for complaints and misconduct, few studies systematically examined them as distinct categories of risk factors, with only two exceptions [50, 65]. One repeatedly validated factor in this realm is the presence of academic/university settings. Factors contributing to this might involve treating patients with complex diagnoses, additional research, teaching and administration responsibilities, and complex organizational structures [55].

Another noteworthy finding is that the issue of complaints or misconduct itself has emerged as a risk factor, suggesting that specific issues can indicate different levels of risk. While this aspect was not extensively emphasized in eligible research, complaint issues have proven significant in identifying and predicting complaints and misconduct risks in practical predictive tools. Our findings reveal that two identified predicting tools incorporate complaint types as indicators—the CPPA PARS system profiles complaint types to reflect complaint distribution related to individuals [55]. Conversely, the PRONE-HP score system provides a summary of the overall complaint-type distribution among health practitioners, revealing strong associations between complaints related to mental health, substance use, sexual boundaries, and honesty with recurrence [1]. However, these models have not integrated external system or environmental factors, making it a potential area for further explorations.

### Strengths and limitations

To our knowledge, this study is the first comprehensive review that synthesizes the issue of risk identification and prediction in the broad field of health practitioners. This expansive research scope enabled us to acquire a thorough understanding of all medico-legal matters related to complaints and misconduct, thereby showcasing a comprehensive landscape within the relevant field. The inclusion of all health practitioners allowed us to gain insights into current research patterns and identify sub-areas that require further exploration. Moreover, this study goes beyond the research progress but also elucidates the practical application, enriching the significant practical value of this research.

However, this study has a few limitations to consider when interpreting the results. Firstly, the language and publication date restrictions may have resulted in missing studies from non-English speaking countries as well as older studies, potentially limiting the scope of the review. Additionally, using a rapid review approach may have introduced some bias into the review process. However, the involvement of the two experts in all screening and assessment processes as much as possible helped to mitigate this potential bias to some extent.

### Interpretation within the context of the wider literature

The findings of this study are in alignment with previous review articles regarding some category of risk factors associated with complaints or misconduct [10, 11]. A systematic review identified risk factors associated with malpractice claims and impaired performance in medical practitioners and categorized these factors as demographic and workplace related, encompassing practice-related elements such as training location, speciality as well as system, organization, and environmental factors related to clinical workload, clinical practice setting, and geographical location [10]. Another focused mapping review synthesized the priori risk factors associated with medical practitioners, placing emphasis on the influence of “human factors” and system factors [11]. This study expands the findings of existing reviews by highlighting the predictive function of complaints/misconduct issues, which have received little attention and were not explicitly suggested until 2019 [1].

In practice, most existing techniques used to analyse patient complaints are complex and pose challenges when implemented in frontline supervision. This is largely due to the lack of technical capacity among regulatory agencies [83]. While the two tools developed so far have addressed this issue to some extent, they still come with some limitations, as both were primarily tailored for specific contexts, limiting their broader applicability. Furthermore, these models do not incorporate system and environment factors. Regulators may face complexity when trying to evaluate these factors across their entire caseload, making them unsuitable candidates for routine-use risk calculators. Given the importance of these contextual factors, future investigations should explore strategies to include them as variables in practical risk measurement [83].

### Implications for policy, practice, and research

This study offers valuable insights for policymakers, educators, regulators, and practitioners, shedding light on the risk areas that need to be addressed, enabling the development of targeted regulatory and educational strategies. The findings also point towards several directions for future research. While robust evidence has been generated from quantitative analysis, we believe that incorporating qualitative approaches would be beneficial in gaining a deeper understanding of the nature, meaning, and mechanisms underlying high-risk and predictive characteristics of complaint and misconduct cases. In addition, the complexity observed in this study and previous research highlights the need to explore the interrelationships among these factors and the potential underlying mechanisms in different contexts. Furthermore, developing more comprehensive and user-friendly predictive tools that can convert research findings into a scoring system to guide interventions is essential. Testing the effectiveness of current prediction models in diverse health settings might also be likely to benefit from refining these predictive tools in practice applications.

## Conclusion

This scoping review advances our understanding of the risk identification and prediction of complaints and misconduct against health practitioners. The findings highlight currently understudied areas, particularly related to qualitative analysis of risk factors, the indicative roles of complaint issues, and the development of practical predictive tools. Furthermore, predicting the complaint risk is multifactorial, non-linear, and context specific. Future in-depth explorations in specific areas will significantly contribute to developing more proactive and data-driven regulations.

## Supplementary Material

mzad114_Supp

## Data Availability

Data relevant to the study are included in the article or uploaded as supplemental information.

## References

[1] Spittal MJ, Bismark MM, Studdert DM. Identification of practitioners at high risk of complaints to health profession regulators. *BMC Health Serv Res* 2019;19:380. 10.1186/s12913-019-4214-y PMC6567559 31196074

[2] Ryan AT, Too LS, Bismark MM. Complaints about chiropractors, osteopaths, and physiotherapists: a retrospective cohort study of health, performance, and conduct concerns. *Chiropr Man Therap* 2018;26:12. 10.1186/s12998-018-0180-4 PMC5896144 29682278

[3] Mattarozzi K, Sfrisi F, Caniglia F et al. What patients’ complaints and praise tell the health practitioner: implications for health care quality. A qualitative research study. *Int J Qual Health Care* 2017;29:83–9. 10.1093/intqhc/mzw139 27920247

[4] Fathy CA, Pichert JW, Domenico H et al. Association between ophthalmologist age and unsolicited patient complaints. *JAMA Ophthalmol* 2018;136:61–7. 10.1001/jamaophthalmol.2017.5154 29192303 PMC5833603

[5] Bismark MM, Fletcher M, Spittal MJ et al. A step towards evidence-based regulation of health practitioners. *Aust Health Rev* 2015;39:483–5. 10.1071/AH14222 25796534

[6] Millbank J . Serious misconduct of health professionals in disciplinary tribunals under the National Law 2010–17. *Aust Health Rev* 2020;44:190–9. 10.1071/AH18239 31671287

[7] Bismark MM, Spittal MJ, Gurrin LC et al. Identification of doctors at risk of recurrent complaints: a national study of healthcare complaints in Australia. *BMJ Qual Saf* 2013;22:532–40. 10.1136/bmjqs-2012-001691 PMC3711360 23576774

[8] Studdert DM, Bismark MM, Mello MM et al. Prevalence and characteristics of physicians prone to malpractice claims. *N Engl J Med* 2016;374:354–62. 10.1056/NEJMsa1506137 26816012

[9] Dambrino RJ, Zuckerman SL, Guidry BS et al. Do neurosurgeons receive more patient complaints than other physicians? Describing who is most at risk and how we can improve. *J Neurosurg* 2021;134:1990–7. 10.3171/2020.4.JNS20870 32736349

[10] Austin EE, Do V, Nullwala R et al. Systematic review of the factors and the key indicators that identify doctors at risk of complaints, malpractice claims or impaired performance. *BMJ Open* 2021;11:e050377. 10.1136/bmjopen-2021-050377 PMC8386219 34429317

[11] Croft E, Clark MT, Efstathiou N et al. A focused mapping review and synthesis of a priori risk factors associated with medical misconduct. *BMJ Open Qual* 2019;8:e000538. 10.1136/bmjoq-2018-000538 PMC6606077 31321315

[12] Bratland SZ, Baste V, Steen K et al. Physician factors associated with increased risk for complaints in primary care emergency services: a case–control study. *BMC Fam Pract* 2020;21:201. 10.1186/s12875-020-01272-0 PMC7519491 32977768

[13] Arksey H, O’Malley L. Scoping studies: towards a methodological framework. *Int J Soc Res Methodol* 2005;8:19–32. 10.1080/1364557032000119616

[14] Levac D, Colquhoun H, O’Brien KK. Scoping studies: advancing the methodology. *Implement Sci* 2010;5:69. 10.1186/1748-5908-5-69 PMC2954944 20854677

[15] Munn Z, Peters MDJ, Stern C et al. Systematic review or scoping review? Guidance for authors when choosing between a systematic or scoping review approach. *BMC Med Res Methodol* 2018;18:143. 10.1186/s12874-018-0611-x PMC6245623 30453902

[16] Tricco AC, Lillie E, Zarin W et al. PRISMA extension for Scoping Reviews (PRISMA-ScR): checklist and explanation. *Ann Intern Med* 2018;169:467–73. 10.7326/M18-0850 30178033

[17] Peters MDJ Godfrey C McInerney P et al. Chapter 11: Scoping Reviews (2020 version). In: Aromataris E and Munn Z (eds), *JBI Manual for Evidence Synthesis*. JBI, 2020. 10.46658/JBIMES-20-12

[18] Heeren P, Hendrikx A, Ceyssens J et al. Structure and processes of emergency observation units with a geriatric focus: a scoping review. *BMC Geriatr* 2021;21:95. 10.1186/s12877-021-02029-9 PMC7852183 33526029

[19] Shemilt I, Khan N, Park S et al. Use of cost-effectiveness analysis to compare the efficiency of study identification methods in systematic reviews. *Syst Rev* 2016;5:140. 10.1186/s13643-016-0315-4 PMC4989498 27535658

[20] Tricco AC, Antony J, Zarin W et al. A scoping review of rapid review methods. *BMC Med* 2015;13:224. 10.1186/s12916-015-0465-6 PMC4574114 26377409

[21] Waffenschmidt S, Knelangen M, Sieben W et al. Single screening versus conventional double screening for study selection in systematic reviews: a methodological systematic review. *BMC Med Res Methodol* 2019;19:132. 10.1186/s12874-019-0782-0 PMC6599339 31253092

[22] Austin EE, Blakely B, Tufanaru C et al. Strategies to measure and improve emergency department performance: a scoping review. *Scand J Trauma Resusc Emerg Med* 2020;28:55. 10.1186/s13049-020-00749-2 PMC7296671 32539739

[23] Lyu S-Y, Liao C-K, Chang K-P et al. Analysis of medical litigation among patients with medical disputes in cosmetic surgery in Taiwan. *Aesthetic Plast Surg* 2011;35:764–72. 10.1007/s00266-011-9684-1 21416296

[24] Sachdeo A, Konfino S, Icyda RU et al. An analysis of patient grievances in a dental school clinical environment. *J Dent Educ* 2012;76:1317–22. 10.1002/j.0022-0337.2012.76.10.tb05386.x 23066130

[25] Kynes JM, Schildcrout JS, Hickson GB et al. An analysis of risk factors for patient complaints about ambulatory anesthesiology care. *Anesth Analg* 2013;116:1325–32. 10.1213/ANE.0b013e31827aef83 23385054

[26] Tessler MJ, Shrier I, Steele RJ. Association between anesthesiologist age and litigation. *Anesthesiology* 2012;116:574–9. 10.1097/ALN.0b013e3182475ebf 22354239

[27] Birkeland S, Depont Christensen R, Damsbo N et al. Characteristics of complaints resulting in disciplinary actions against Danish GPs. *Scand J Prim Health Care* 2013;31:153–7. 10.3109/02813432.2013.823768 23906082 PMC3750437

[28] McGregor MJ, Cohen M, Stocks-Rankin C-R et al. Complaints in for-profit, non-profit and public nursing homes in two Canadian provinces. *Open Med* 2011;5:e183–92. 22567074 PMC3345377

[29] Baker SR, Whang JS, Luk L et al. The demography of medical malpractice suits against radiologists. *Radiology* 2013;266:539–47. 10.1148/radiol.12110971 23192777

[30] Nikoghosyan-Bossen G 1, Hauberg A, Homøe P 1. Increased number of ear-nose-throat malpractice complaints in Denmark. *Dan Med J* 2012;59:1–5. 22549483

[31] Guidera M, McCool W, Hanlon A et al. Midwives and liability: results from the 2009 nationwide survey of certified nurse-midwives and certified midwives in the United States. *J Midwifery Womens Health* 2012;57:345–52. 10.1111/j.1542-2011.2012.00201.x 22758356

[32] Birkeland S, Christensen RD, Damsbo N et al. Patient complaint cases in primary health care: what are the characteristics of general practitioners involved? *Biomed Res Int* 2013;2013:1–5. 10.1155/2013/807204 PMC3763590 24027764

[33] Phipps DL, Noyce PR, Walshe K et al. Pharmacists subjected to disciplinary action: characteristics and risk factors. *Int J Pharm Pract* 2011;19:367–73. 10.1111/j.2042-7174.2011.00119.x 21899618

[34] Bismark MM, Spittal MJ, Studdert DM. Prevalence and characteristics of complaint‐prone doctors in private practice in Victoria. *Med J Aust* 2011;195:25–8. 10.5694/j.1326-5377.2011.tb03183.x 21728937

[35] Samenow CP, Yabiku ST, Ghulyan M et al. The role of family of origin in physicians referred to a CME course. *HEC Forum* 2012;24:115–26. 10.1007/s10730-011-9171-8 22113587

[36] Mehtsun WT, Ibrahim AM, Diener-West M et al. Surgical never events in the United States. *Surgery* 2013;153:465–72. 10.1016/j.surg.2012.10.005 23257079

[37] Gogos AJ, Clark RB, Bismark MM et al. When informed consent goes poorly: a descriptive study of medical negligence claims and patient complaints. *Med J Aust* 2011;195:340–4. 10.5694/mja11.10379 21929499

[38] Casali MB, Blandino A, Del Sordo S et al. Alleged malpractice in orthopaedics. Analysis of a series of medical insurance claims. *J Orthop Traumatol* 2018;19:7. 10.1186/s10195-018-0500-4 PMC6093819 30112637

[39] Pereira-Lima K, Mata DA, Loureiro SR et al. Association between physician depressive symptoms and medical errors: a systematic review and meta-analysis. *JAMA Network Open* 2019;2:e1916097. 10.1001/jamanetworkopen.2019.16097 PMC6902829 31774520

[40] Shouhed D, Beni C, Manguso N et al. Association of emotional intelligence with malpractice claims: a review. *JAMA Surg* 2019;154:250–6. 10.1001/jamasurg.2018.5065 30698614

[41] Welle D, Trockel MT, Hamidi MS et al. Association of occupational distress and sleep-related impairment in physicians with unsolicited patient complaints. *Mayo Clin Proc* 2020;95:719–26. 10.1016/j.mayocp.2019.09.025 32247345

[42] Liu Z, Zhang Y, Asante JO et al. Characteristics of medical disputes arising from dental practice in Guangzhou, China: an observational study. *BMJ Open* 2018;8:e018738. 10.1136/bmjopen-2017-018738 PMC5829771 29439073

[43] Panuganti PL, Hartnett DA, Eltorai AEM et al. Colorectal cancer litigation: 1988–2018. *Am J Gastroenterol* 2020;115:1525–31. 10.14309/ajg.0000000000000705 32453040

[44] Veness BG, Tibble H, Grenyer BF et al. Complaint risk among mental health practitioners compared with physical health practitioners: a retrospective cohort study of complaints to health regulators in Australia. *BMJ Open* 2019;9:e030525. 10.1136/bmjopen-2019-030525 PMC7008450 31874871

[45] Thomas LA, Tibble H, Too LS et al. Complaints about dental practitioners: an analysis of 6 years of complaints about dentists, dental prosthetists, oral health therapists, dental therapists and dental hygienists in Australia. *Aust Dent J* 2018;63:285–93. 10.1111/adj.12625 29862517 PMC6635754

[46] Birkeland S, Bismark M, Barry MJ et al. Does greater patient involvement in healthcare decision-making affect malpractice complaints? A large case vignette survey. *PLoS One* 2021;16:e0254052. 10.1371/journal.pone.0254052 PMC8253406 34214136

[47] De Champlain AF, Ashworth N, Kain N et al. Does pass/fail on medical licensing exams predict future physician performance in practice? *J Med Regul* 2020;106:17–26. 10.30770/2572-1852-106.4.17

[48] Birkeland S, Bogh SB, Cheng M. Education trajectories and malpractice complaints—a study among Danish general practitioners. *Cogent Educ* 2018;5:1473747. 10.1080/2331186X.2018.1473747

[49] Schaffer AC, Babayan A, Yu-Moe CW et al. The effect of clinical volume on annual and per-patient encounter medical malpractice claims risk. *J Patient Saf* 2021;17:e995–1000. 10.1097/PTS.0000000000000706 32209950

[50] Birkeland S, Bogh SB. General practice location and malpractice litigation. *Rural Remote Health* 2019;19:4663. 10.22605/RRH4663 30797227

[51] Walton M, Kelly PJ, Chiarella EM et al. Profile of the most common complaints for five health professions in Australia. *Aust Health Rev* 2020;44:15–23. 10.1071/AH18074 31658934

[52] Carlson JN, Foster KM, Pines JM et al. Provider and practice factors associated with emergency physicians’ being named in a malpractice claim. *Ann Emerg Med* 2018;71:157–164.e4. 10.1016/j.annemergmed.2017.06.023 28754358 PMC5785561

[53] Schaffer AC, Yu-Moe CW, Babayan A et al. Rates and characteristics of medical malpractice claims against hospitalists. *J Hosp Med* 2021;16:390–6. 10.12788/jhm.3557 34197302

[54] Nassiri AM, Pichert JW, Domenico HJ et al. Unsolicited patient complaints among otolaryngologists. *Otolaryngol Head Neck Surg* 2019;160:810–7. 10.1177/0194599818823706 30642235

[55] Raldow A, Adefres B, Warso M et al. Unsolicited patient complaints among radiation, medical, and surgical oncologists. *Cancer* 2021;127:2350–7. 10.1002/cncr.33513 33724453

[56] Tibble HM, Broughton NS, Studdert DM et al. Why do surgeons receive more complaints than their physician peers? Complaints about surgeons and physicians. *ANZ J Surg* 2018;88:269–73. 10.1111/ans.14225 28889480

[57] Menon NK, Shanafelt TD, Sinsky CA et al. Association of physician burnout with suicidal ideation and medical errors. *JAMA Network Open* 2020;3:e2028780. 10.1001/jamanetworkopen.2020.28780 PMC7726631 33295977

[58] Stimson CJ, Pichert WJ, Ilene NM et al. Medical malpractice claims risk in urology: an empirical analysis of patient complaint data. *J Urol* 2010;183:1971–6. 10.1016/j.juro.2010.01.027 20303531

[59] Dallal RM, Pang J, Soriano I et al. Bariatric-related medical malpractice experience: survey results among ASMBS members. *Surg Obes Relat Dis* 2014;10:121–4. 10.1016/j.soard.2013.04.015 24054470

[60] Kohatsu ND, Gould D, Ross LK et al. Characteristics associated with physician discipline: a case-control study. *Arch Intern Med* 2004;164:653–8. 10.1001/archinte.164.6.653 15037494

[61] Clay SW, Conatser RR. Characteristics of physicians disciplined by the State Medical Board of Ohio. *J Am Osteopath Assoc* 2003;103:81–8. 12622353

[62] Khaliq AA, Dimassi H, Huang CY et al. Disciplinary action against physicians: who is likely to get disciplined? *Am J Med* 2005;118:773–7. 10.1016/j.amjmed.2005.01.051 15989912

[63] Papadakis MA, Teherani A, Banach MA et al. Disciplinary action by medical boards and prior behavior in medical school. *N Engl J Med* 2005;353:2673–82. 10.1056/NEJMsa052596 16371633

[64] Virapongse A, Bates DW, Shi P et al. Electronic health records and malpractice claims in office practice. *Arch Intern Med* 2008;168:2362–7. 10.1001/archinte.168.21.2362 19029502

[65] Rolph JE, Adams JL, McGuigan KA. Identifying malpractice-prone physicians. *J Empir Leg Stud* 2007;4:125–53. 10.1111/j.1740-1461.2007.00084.x

[66] Perlis CS, Campbell RM, Perlis RH et al. Incidence of and risk factors for medical malpractice lawsuits among Mohs surgeons. *Dermatol Surg* 2006;32:79–83. 10.1097/00042728-200601000-00016 16393602

[67] Abbott RL, Ou RJ, Bird M. Medical malpractice predictors and risk factors for ophthalmologists performing LASIK and photorefractive keratectomy surgery. *Ophthalmology* 2003;110:2137–46. 10.1016/j.ophtha.2003.07.001 14597521

[68] Weycker DA, Jensen GA. Medical malpractice among physicians: who will be sued and who will pay? *Health Care Manag Sci* 2000;3:269–77. 10.1023/A:1019014028914 11105413

[69] Mangalmurti S, Seabury SA, Chandra A et al. Medical professional liability risk among US cardiologists. *Am Heart J* 2014;167:690–6. 10.1016/j.ahj.2014.02.007 24766979 PMC4153384

[70] Waters TM, Lefevre FV, Budetti PP. Medical school attended as a predictor of medical malpractice claims. *Qual Saf Health Care* 2003;12:330–6. 10.1136/qhc.12.5.330 14532363 PMC1743759

[71] Hickson GB, Federspiel CF, Pichert JW et al. Patient complaints and malpractice risk. *JAMA* 2002;287:2951–7. 10.1001/jama.287.22.2951 12052124

[72] Resnick AS, Mullen JL, Kaiser LR et al. Patterns and predictions of resident misbehavior—a 10-year retrospective look. *Curr Surg* 2006;63:418–25. 10.1016/j.cursur.2006.05.004 17084771

[73] Papadakis MA, Arnold GK, Blank LL et al. Performance during internal medicine residency training and subsequent disciplinary action by state licensing boards. *Ann Intern Med* 2008;148:869–76. 10.7326/0003-4819-148-11-200806030-00009 18519932

[74] Jena AB, Schoemaker L, Bhattacharya J et al. Physician spending and subsequent risk of malpractice claims: observational study. *BMJ* 2015;351:h5516. 10.1136/bmj.h5516 PMC4633452 26538498

[75] Chauhan SP, Chauhan VB, Cowan BD et al. Professional liability claims and Central Association of Obstetricians and Gynecologists members: myth versus reality. *Am J Obstet Gynecol* 2005;192:1820–6. 10.1016/j.ajog.2004.12.058 15970818

[76] Kohanim S, Sternberg P, Karrass J et al. Unsolicited patient complaints in ophthalmology: an empirical analysis from a large national database. *Ophthalmology* 2016;123:234–41. 10.1016/j.ophtha.2015.10.010 26589549

[77] Papadakis MA, Hodgson CS, Teherani A et al. Unprofessional behavior in medical school is associated with subsequent disciplinary action by a state medical board. *Acad Med* 2004;79:244–9. 10.1097/00001888-200403000-00011 14985199

[78] Boyll P, Kang P, Mahabir R et al. Variables that impact medical malpractice claims involving plastic surgeons in the United States. *Aesthet Surg J* 2017;38:785–92. 10.1093/asj/sjx182 29040404

[79] Adamson TE, Baldwin DC Jr, Oppenberg A et al. The virtuous orthopaedist has fewer malpractice suits. *Clin Orthop Relat Res* 2000;378:104–9. 10.1097/00003086-200009000-00017 10986982

[80] Taylor DM, Wolfe RS, Cameron PA. Analysis of complaints lodged by patients attending Victorian hospitals, 1997–2001. *Med J Aust* 2004;181:31–5. 10.5694/j.1326-5377.2004.tb06157.x 15233610

[81] Nash LM, Kelly PJ, Daly MG et al. Australian doctors’ involvement in medicolegal matters: a cross-sectional self-report study. *Med J Aust* 2009;191:436–40. 10.5694/j.1326-5377.2009.tb02879.x 19835537

[82] Nash L, Daly M, Johnson M et al. Personality, gender and medico-legal matters in medical practice. *Australas Psychiatry* 2009;17:19–24. 10.1080/10398560802085359 18608158

[83] Spittal MJ, Bismark MM, Studdert DM. The PRONE score: an algorithm for predicting doctors’ risks of formal patient complaints using routinely collected administrative data. *BMJ Qual Saf* 2015;24:360–8. 10.1136/bmjqs-2014-003834 PMC4453507 25855664

[84] Liu JJ, Alam AQ, Goldberg HR et al. Characteristics of internal medicine physicians disciplined by professional colleges in Canada. *Medicine* (*Baltimore)* 2015;94:e937–e937. 10.1097/MD.0000000000000937 26131839 PMC4504618

[85] Tamblyn R, Abrahamowicz M, Dauphinee D et al. Physician scores on a national clinical skills examination as predictors of complaints to medical regulatory authorities. *JAMA* 2007;298:993–1001. 10.1001/jama.298.9.993 17785644

[86] Ambady N, LaPlante D, Nguyen T et al. Surgeons’ tone of voice: a clue to malpractice history. *Surgery* 2002;132:5–9. 10.1067/msy.2002.124733 12110787

[87] Yates J, and James D. Risk factors at medical school for subsequent professional misconduct: multicentre retrospective case-control study. *BMJ* 2010;340:c2040. 10.1136/bmj.c2040 PMC3191727 20423965

[88] Otaki Y, Ishida MD, Saito Y et al. Analysis of closed claims in the clinical management of rheumatoid arthritis in Japan. *Chin Med J Engl* 2017;130:1454–8. 10.4103/0366-6999.207479 28584209 PMC5463476

[89] Unwin E, Woolf K, Wadlow C et al. Disciplined doctors: does the sex of a doctor matter? A cross-sectional study examining the association between a doctor’s sex and receiving sanctions against their medical registration. *BMJ Open* 2014;4:e005405. 10.1136/bmjopen-2014-005405 PMC4127941 25104057

[90] Wu CY, Lai HJ, Chen RC. Medical malpractice experience of Taiwan: 2005 versus 1991. *Intern Med J* 2009;39:237–42. 10.1111/j.1445-5994.2009.01801.x 19402862

[91] Wu CY, Lai HJ, Chen RC. Patient characteristics predict occurrence and outcome of complaints against physicians: A study from a medical center in central Taiwan. *J Formos Med Assoc* 2009;108:126–34. 10.1016/S0929-6646(09)60043-7 19251548

[92] Tsimtsiou Z, Kirana PS, Hatzimouratidis K et al. What is the profile of patients thinking of litigation? Results from the hospitalized and outpatients’ profile and expectations study. *Hippokratia* 2014;18:139–43. 25336877 PMC4201400

[93] Cunningham W, Crump R, and Tomlin A. The characteristics of doctors receiving medical complaints: a cross-sectional survey of doctors in New Zealand. *N Z Med J* 2003;116:1183. 14581939

[94] Unwin E, Woolf K, Wadlow C et al. Sex differences in medico-legal action against doctors: a systematic review and meta-analysis. *BMC Med* 2015;13:172–172. 10.1186/s12916-015-0413-5 26268807 PMC4535538

[95] Joffe AM, Aziz MF, Posner KL et al. Management of difficult tracheal intubation: a closed claims analysis. *Anesthesiology* 2019;131:818–29. 10.1097/ALN.0000000000002815 31584884 PMC6779339

