# Supplementary material for: Risk identification and prediction of complaints and misconduct against health practitioners: a scoping review
Source: Int J Qual Health Care. 2023 Dec 29;36(1):mzad114. doi: 10.1093/intqhc/mzad114 (PMC10791111; doi:10.1093/intqhc/mzad114)
Supplement: mzad114_Supp [file mzad114_supp.zip › suppl_data/Supplementary material.pdf]

Appendix A. Full search strategy

Database	Searches
**Pubmed**<br>**(n=1771)**	("health personnel"[MeSH Terms] OR "health personnel*"[Title/Abstract] OR "health care personnel*"[Title/Abstract] OR "health practitioner*"[Title/Abstract] OR "health professional*"[Title/Abstract] OR "pharmacist*"[Title/Abstract] OR "physician*" [Title/Abstract] OR "doctor*"[Title/Abstract] OR "nurse*"[Title/Abstract]) **AND** ("complaint*"[Title/Abstract] OR "misconduct*"[Title/Abstract] OR "malpractice*"[Title/Abstract] OR "malpractice"[MeSH Terms]) **AND** ("characteristic*"[Title/Abstract] OR "predictor*"[Title/Abstract] OR "risk factor*"[Title/Abstract] OR "taxonomy"[Title/Abstract] OR "taxonomies"[Title/Abstract] OR "classification*"[Title/Abstract] OR "classification"[MeSH Terms])<br>Filters: English, from 2000 - 2021
**EMBASE**<br>**(n=2768)**	1. *health care personnel/ or *health practitioner/ or *pharmacist/ or *physician/ or *nurse/<br>2. (health personnel* or health care personnel* or health practitioner* or health professional* or pharmacist* or doctor* or physician* or nurse*).ab,kw,ti.<br>3. 1 or 2<br>4. *malpractice/<br>5. (complaint* or misconduct* or malpractice*).ab,kw,ti.<br>6. 4 or 5<br>7. *risk factor/ or *taxonomy/ or *classification/<br>8. (characteristic* or predictor* or risk factor* or taxonomy or taxonomies or classification*).ab,kw,ti.<br>9. 7 or 8<br>10. 3 and 6 and 9<br>11. limit 10 to (english language and yr="2000 -Current")
**CINAHL Plus**<br>**(n=934)**	(TI ("health personnel*" OR "health care personnel*" OR "health practitioner*" OR "health professional*" OR "pharmacist*" OR "physician*" OR "doctor*" OR "nurse*") OR AB ("health personnel*" OR "health care personnel*" OR "health practitioner*" OR "health professional*" OR "pharmacist*" OR "physician*" OR "doctor*" OR "nurse*") OR MH ("health personnel*" OR "health care personnel*" OR "health practitioner*" OR "health professional*" OR "pharmacist*" OR "physician*" OR "doctor*" OR "nurse*")) **AND** (TI ("complaint*" OR "misconduct*" OR "malpractice*") OR AB ("complaint*" OR "misconduct*" OR "malpractice*") OR MH ("complaint*" OR "misconduct*" OR "malpractice*")) **AND** (TI ("characteristic*" OR "predictor*" OR "risk factor*" OR "taxonomy" OR "taxonomies" OR "classification*") OR AB ("characteristic*" OR "predictor*" OR "risk factor*" OR "taxonomy" OR "taxonomies" OR "classification*") OR MH ("characteristic*" OR "predictor*" OR "risk factor*" OR "taxonomy" OR "taxonomies" OR "classification*"))<br>**Limiters** - Publication Year: 2000-; English Language<br>**Expanders** - Apply equivalent subjects<br>**Search modes** - Find all my search terms

Appendix B. Basic information of eligible studies

ID #	Author & Year[Reference#]	Title	Journal	Publication Year	Location	Study Design	Data Source	Year of Study Conducted	Involved Profession(s)
8	Casali 2018[38]	Alleged malpractice in orthopaedics. Analysis of a series of medmal insurance claims	Journal of Orthopaedics and Traumatology	2018	Italy	Quantitative	Insurer	2002-2013	Physicians
11	Otaki 2017[88]	Analysis of closed claims in the clinical management of rheumatoid arthritis in Japan	Chinese Medical Journal	2017	Japan	Quantitative	Insurer	2004-2014	Unspecified
12	Taylor 2004[80]	Analysis of complaints lodged by patients attending Victorian hospitals, 1997-2001	The Medical journal of Australia	2004	Australia	Quantitative	Regulatory Agency	1997-2001	Unspecified
17	Lyu 2011[23]	Analysis of medical litigation among patients with medical disputes in cosmetic surgery in Taiwan	Aesthetic plastic surgery	2011	Taiwan, China	Quantitative	Health Facility	2001-2009	Physicians
20	Sachdeo 2012[24]	An Analysis of Patient Grievances in a Dental School Clinical Environment	Journal of Dental Education	2012	the United States(USA)	Quantitative	Health Facility	2005-2008	Dentists
22	Kynes 2013[25]	An analysis of risk factors for patient complaints about ambulatory anesthesiology care	Anesthesia and Analgesia	2013	the United States(USA)	Quantitative	Academic Medical Center	2006-2010	Physicians
24	Tessler 2012[26]	Association between Anesthesiologist Age and Litigation	Anesthesiology	2012	Canada	Quantitative	Regulatory Agency	1993-2002	Physicians
25	Fathy 2018[4]	Association Between Ophthalmologist Age and Unsolicited Patient Complaints	JAMA Ophthalmology	2018	the United States(USA)	Quantitative	Academic Medical Center	2002-2015	Physicians
26	Pereira-Lima 2019[39]	Association Between Physician Depressive Symptoms and Medical Errors: A Systematic Review and Meta-analysis	JAMA Network Open	2019	Other	Review	Literature	Before 2018	Physicians
29	Shouhed 2019[40]	Association of Emotional Intelligence With Malpractice Claims: A Review	JAMA Surgery	2019	Other	Review	Literature	2017	Physicians
30	Welle 2020[41]	Association of Occupational Distress and Sleep-Related Impairment in Physicians With Unsolicited Patient Complaints	Mayo Clinic Proceedings	2020	the United States(USA)	Quantitative	Survey	2013-2016	Physicians
31	Menon 2020[57]	Association of Physician Burnout With Suicidal Ideation and Medical Errors	JAMA Network Open	2020	the United States(USA)	Quantitative	Survey	2018-2019	Physicians
34	Nash 2009[81]	Australian doctors' involvement in medicolegal matters: a cross-sectional self-report study	Medical Journal of Australia	2009	Australia	Quantitative	Survey	2007	Doctors

36	Dallal 2014[59]	Bariatric-related medical malpractice experience:survey results among ASMBS members	Surgery for Obesity and Related Diseases	2014	the United States(USA)	Quantitative	Survey	2011	Physicians
45	Kohatsu 2004[60]	Characteristics Associated With Physician Discipline: a case-control study	Archives of Internal Medicine	2004	the United States(USA)	Quantitative	Regulatory Agency	1998-2001	Physicians
46	Birkeland 2013[27]	Characteristics of complaints resulting in disciplinary actions against Danish GPs	Scandinavian Journal of Primary Health Care	2013	Denmark	Quantitative	Regulatory Agency	2007	Physicians
47	Cunningham 2003[93]	The characteristics of doctors receiving medical complaints: a cross-sectional survey of doctors in New Zealand	New Zealand Medical Journal	2003	New Zealand	Mixed	Survey	2001	Doctors
48	Liu 2015[84]	Characteristics of Internal Medicine Physicians Disciplined by Professional Colleges in Canada	Open Medicine	2015	Canada	Quantitative	Industry Association	2000-2013	Physicians
49	Liu 2018[42]	Characteristics of medical disputes arising from dental practice in Guangzhou, China: An observational study	BMJ Open	2018	Mainland China	Quantitative	Survey	2008-2012	Dentists
61	Clay 2003[61]	Characteristics of physicians disciplined by the State Medical Board of Ohio	The Journal of the American Osteopathic Association	2003	the United States(USA)	Quantitative	Regulatory Agency	1997-1999	Physicians
72	Panuganti 2020[43]	Colorectal Cancer Litigation: 1988-2018	The American Journal of Gastroenterology	2020	the United States(USA)	Quantitative	For-Profit Online Database	1988-2018	Physicians
76	Veness 2019[44]	Complaint risk among mental health practitioners compared with physical health practitioners: A retrospective cohort study of complaints to health regulators in Australia	BMJ Open	2019	Australia	Quantitative	Regulatory Agency	2011-2016	Physicians
77	Ryan 2018[2]	Complaints about chiropractors, osteopaths, and physiotherapists: a retrospective cohort study of health, performance, and conduct concerns	Chiropractic & Manual Therapies	2018	Australia	Quantitative	Regulatory Agency	2011-2016	Physicians
78	Thomas 2018[45]	Complaints about dental practitioners: an analysis of 6 years of complaints about dentists, dental prosthetists, oral health therapists, dental therapists and dental hygienists in Australia	Australian Dental Journal	2018	Australia	Quantitative	Regulatory Agency	2011-2016	Dentists
83	McGregor 2011[28]	Complaints in for-profit, non-profit and public nursing homes in two Canadian provinces	Open medicine	2011	Canada	Quantitative	Regulatory Agency, Health Facilities, Online Database	2004-2008	Unspecified
98	Baker 2013[29]	The Demography of Medical Malpractice Suits against Radiologists	Radiology	2013	the United States(USA)	Quantitative	Regulatory Agency, Self-Reported	2007-2010	Physicians

110	Khaliq 2005[62]	Disciplinary action against physicians: Who is likely to get disciplined?	American Journal of Medicine	2005	the United States(USA)	Quantitative	Regulatory Agency	2001	Physicians
111	Papadakis 2005[63]	Disciplinary action by medical boards and prior behavior in medical school	New England Journal of Medicine	2005	the United States(USA)	Quantitative	Regulatory Agency	1990-2003	Physicians
112	Unwin 2014[89]	Disciplined doctors: Does the sex of a doctor matter? A cross-sectional study examining the association between a doctor's sex and receiving sanctions against their medical registration	BMJ open	2014	United Kingdom(UK)	Quantitative	Regulatory Agency	2005-2013	Doctors
114	Dambrino 2021[9]	Do neurosurgeons receive more patient complaints than other physicians? Describing who is most at risk and how we can improve	Journal of Neurosurgery	2021	the United States(USA)	Quantitative	Academic Medical Center	2014-2017	Physicians
122	Birkeland 2021[46]	Does greater patient involvement in healthcare decision-making affect malpractice complaints?A large case vignette survey	PLOS ONE	2021	Denmark	quantitative	survey	2019	Unspecified
123	DeChamplain 2020[47]	Does Pass/Fail on Medical Licensing Exams Predict Future Physician Performance in Practice? A Longitudinal Cohort Study of Alberta Physicians	Journal of Medical Regulation	2020	Canada	Quantitative	Industry Association	1992-2017	Physicians
124	Birkeland 2018[48]	Education trajectories and malpractice complaints—A study among Danish general practitioners	Cogent Education	2018	Denmark	Quantitative	Regulatory Agency	2007	Physicians
125	Schaffer 2020[49]	The Effect of Clinical Volume on Annual and Per-Patient Encounter Medical Malpractice Claims Risk	Journal of Patient Safety	2020	the United States(USA)	Quantitative	Insurer	2010-2014	Physicians
126	Virapongse 2008[64]	Electronic health records and malpractice claims in office practice	Archives of Internal Medicine	2008	the United States(USA)	Quantitative	Survey	2005	Physicians
136	Croft 2019[11]	A focused mapping review and synthesis of a priori risk factors associated with medical misconduct	BMJ Open Quality	2019	Other	Review	Regulatory Agency	2016-2017	Doctors
139	Birkeland 2019[50]	General practice location and malpractice litigation	Rural Remote Health	2019	Denmark	Quantitative	Regulatory Agency	2007	Physicians
148	Bismark 2013[7]	Identification of doctors at risk of recurrent complaints: A national study of healthcare complaints in Australia	BMJ Quality and Safety	2013	Australia	Quantitative	Regulatory Agency	2011-2012	Doctors
149	Spittal 2019[1]	Identification of practitioners at high risk of complaints to health profession regulators	BMC Health Services Research	2019	Australia	Mixed	Regulatory Agency	2011-2016	Health Practitioners (include pharmacists)
150	Rolph 2007[65]	Identifying Malpractice-Prone Physicians	Journal of Empirical Legal Studies	2007	the United States(USA)	Quantitative	Insurer	1977-1989	Physicians

153	Perlis 2006[66]	Incidence of and risk factors for medical malpractice lawsuits among Mohs surgeons	Dermatologic Surgery	2006	the United States(USA)	Quantitative	Survey	2004	Physicians
154	Nikoghosyan-Bossen 2012[30]	Increased number of ear-nose-throat malpractice complaints in Denmark	Danish Medical Bulletin	2012	Denmark	Quantitative	Regulatory Agency	1998-2008	Physicians
188	Weycker 2000[68]	Medical malpractice among physicians: who will be sued and who will pay?	Health Care Management Science	2000	the United States(USA)	Quantitative	Insurer	1980-1989	Physicians
196	Stimson 2010[58]	Medical Malpractice Claims Risk in Urology: An Empirical Analysis of Patient Complaint Data	Journal of Urology	2010	the United States(USA)	Quantitative	Academic Medical Center	2004-2007	Physicians
197	Wu 2009[90]	Medical malpractice experience of Taiwan: 2005 versus 1991	Internal Medicine Journal	2009	Taiwan, China	Quantitative	Survey	1991-2005	Physicians
208	Abbott 2003[67]	Medical Malpractice Predictors and Risk Factors for Ophthalmologists Performing LASIK and Photorefractive Keratectomy Surgery	American Academy of Ophthalmology	2003	the United States(USA)	Quantitative	Insurer	1996-2002	Physicians
211	Mangalmurti 2014[69]	Medical professional liability risk among US cardiologists	American Heart Journal	2014	the United States(USA)	Quantitative	Insurer	1991-2005	Physicians
212	Waters 2003[70]	Medical school attended as a predictor of medical malpractice claims	Quality & Safety in Health Care	2003	the United States(USA)	Quantitative	Industry Association	1990-1997	Physicians
221	Guidera 2012[31]	Midwives and Liability: Results from the 2009 Nationwide Survey of Certified Nurse-Midwives and Certified Midwives in the United States	Journal of Midwifery & Women's Health	2012	the United States(USA)	Quantitative	Survey	2009	Nurses
240	Wu 2009[91]	Patient characteristics predict occurrence and outcome of complaints against physicians: A study from a medical center in central Taiwan	Journal of the Formosan Medical Association	2009	Taiwan, China	Quantitative	Health Facility	1991-2005	Physicians
241	Birkeland 2013[32]	Patient Complaint Cases in Primary Health Care: What Are the Characteristics of General Practitioners Involved?	BioMed Research International	2013	Denmark	Quantitative	Regulatory Agency	2007	Physicians
244	Hickson 2002[71]	Patient Complaints and Malpractice Risk	JAMA: Journal of the American Medical Association	2002	the United States(USA)	Quantitative	Regulatory Agency	1992-1998	Physicians
253	Resnick 2006[72]	Patterns and Predictions of Resident Misbehavior-A 10-Year Retrospective Look	Current Surgery	2006	the United States(USA)	Quantitative	Medical School	1995-2005	Physicians
254	Papadakis 2008[73]	Performance during Internal Medicine Residency Training and Subsequent Disciplinary Action by State Licensing Boards	Annals of Internal Medicine	2008	the United States(USA)	Quantitative	Regulatory Agency	1990-2006	Physicians

255	Nash 2009[82]	Personality, gender and medico-legal matters in medical practice	Australasian Psychiatry	2009	Australia	Quantitative	Survey	2006	Physicians
257	Phipps 2011[33]	Pharmacists subjected to disciplinary action: characteristics and risk factors	International Journal of Pharmacy Practice	2011	United Kingdom(UK)	Quantitative	Regulatory Agency	2007-2009	Pharmacists
258	Bratland 2020[12]	Physician factors associated with increased risk for complaints in primary care emergency services: a case -control study	BMC family practice	2020	Norway	Quantitative	Health Facility	2015-2017	Physicians
261	Tamblyn 2007[85]	Physician scores on a national clinical skills examination as predictors of complaints to medical regulatory authorities	JAMA: Journal of the American Medical Association	2007	Canada	Quantitative	Regulatory Agency	1993-1996	Physicians
262	Jena 2015[74]	Physician spending and subsequent risk of malpractice claims: Observational study	BMJ (Online)	2015	the United States(USA)	Quantitative	Regulatory Agency	2000-2009	Physicians
272	Bismark 2011[34]	Prevalence and characteristics of complaint-prone doctors in private practice in Victoria	Medical Journal of Australia	2011	Australia	Quantitative	Regulatory Agency	2000-2009	Doctors
274	Studdert 2016[8]	Prevalence and Characteristics of Physicians Prone to Malpractice Claims	New England Journal of Medicine	2016	Australia	Quantitative	Regulatory Agency	2005-2014	Physicians
278	Chauhan 2005[75]	Professional liability claims and Central Association of Obstetricians and Gynecologists members: Myth versus reality	American Journal of Obstetrics and Gynecology	2005	the United States(USA)	Quantitative	Industry Association	2003	Physicians
283	Walton 2020[51]	Profile of the most common complaints for five health professions in Australia	Australian Health Review	2020	Australia	Quantitative	Regulatory Agency	2012-2013	Health Practitioners (include pharmacists)
284	Spittal 2015[83]	The PRONE score: an algorithm for predicting doctors' risks of formal patient complaints using routinely collected administrative data	BMJ Quality & Safety	2015	Australia	Quantitative	Regulatory Agency	2000-2011	Doctors
285	Carlson 2018[52]	Provider and Practice Factors Associated With Emergency Physicians' Being Named in a Malpractice Claim	Annals of Emergency Medicine	2018	the United States(USA)	Quantitative	Industry Association	2010-2014	Physicians
289	Schaffer 2021[53]	Rates and Characteristics of Medical Malpractice Claims Against Hospitalists	Journal of hospital medicine	2021	the United States(USA)	Quantitative	Regulatory Agency	2009-2018	Physicians
306	Yates 2010[87]	Risk factors at medical school for subsequent professional misconduct: multicentre retrospective case-control study	BMJ (Online)	2010	United Kingdom(UK)	Quantitative	Medical School, Regulatory Agency	1999-2004	Doctors
310	Samenow 2012[35]	The role of family of origin in physicians referred to a CME course	HEC Forum	2012	United Kingdom(UK)	Quantitative	Survey	2000-2009	Physicians

313	Unwin 2015[94]	Sex differences in medico-legal action against doctors: a systematic review and meta-analysis	BMC Medicine	2015	Other	Review	Literature	Before 2015	Doctors
320	Ambady 2002[86]	Surgeons' tone of voice: A clue to malpractice history	Surgery	2002	Canada	Mixed	Health Facility	Not given	Physicians
323	Mehtsun 2013[36]	Surgical never events in the United States	Surgery	2013	the United States(USA)	Quantitative	Regulatory Agency	1990-2010	Physicians
326	Austin 2021[10]	Systematic review of the factors and the key indicators that identify doctors at risk of complaints, malpractice claims or impaired performance	BMJ Open	2021	Other	Review	Literature	2011-2020	Doctors
335	Papadakis 2004[77]	Unprofessional Behavior in Medical School Is Associated with Subsequent Disciplinary Action by a State Medical Board	Academic Medicine	2004	the United States(USA)	Quantitative	Medical School, Regulatory Agency	1999-2000	Physicians
336	Nassiri 2019[54]	Unsolicited Patient Complaints among Otolaryngologists	Otolaryngology-Head & Neck Surgery	2019	the United States(USA)	Quantitative	Academic Medical Center	2014-2017	Physicians
337	Raldow 2021[55]	Unsolicited patient complaints among radiation, medical, and surgical oncologists	Cancer	2021	the United States(USA)	Quantitative	Academic Medical Center	2015-2018	Physicians
338	Kohanim 2016[76]	Unsolicited Patient Complaints in Ophthalmology	American Academy of Ophthalmology	2016	the United States(USA)	Quantitative	Academic Medical Center	2009-2013	Physicians
341	Boyll 2017[78]	Variables That Impact Medical Malpractice Claims Involving Plastic Surgeons in the United States	Aesthetic Surgery Journal	2017	the United States(USA)	Quantitative	Survey	2017	Physicians
343	Adamson 2000[79]	The virtuous orthopaedist has fewer malpractice suits	Clinical Orthopaedics and Related Research	2000	the United States(USA)	Quantitative	Survey	Not given	Physicians
347	Tsimtsiou 2014[92]	What is the profile of patients thinking of litigation? Results from the hospitalized and outpatients' profile and expectations study	Hippokratia	2014	Greece	Quantitative	Health Facility	Not given	Physicians
349	Gogos 2011[37]	When informed consent goes poorly: a descriptive study of medical negligence claims and patient complaints	Medical Journal of Australia	2011	Australia	Quantitative	Insurer	2002-2008	Doctors
353	Tibble 2018[56]	Why do surgeons receive more complaints than their physician peers?	ANZ journal of surgery	2018	Australia	Quantitative	Regulatory Agency	2011-2016	Physicians

Appendix C. Synthesises on the risk factors

Main Categories	Sub Categories	Risk Factors	ID#
Health Practitioners' Characteristics			
Basic Demographics	Age	Older	24 45 76 77 78 123 148 149 150 197 212 221 274 283 353
		Younger	25 338
	Gender	Male	34 45 47 72 76 77 78 98 110 112 123 126 136 148 149 150 188 197 208 244 255 261 272 274 278 283 284 306 310 313 336 337 353
		Female	338
	Ethnicity	Non-white	110
		White	310
	Place of Birth	Non-Australia born, unless from the UK or Ireland	283
	Social Class	Lower estimated social class	306
	Marital Status	Being partnered or divorced/separated	34 310
	Family Background	From families that were scored in the "extreme" category (disengaged and rigid group)	310
Education Characteristics	Location of Education	Domestic (Australia) graduate	34 272
		International graduate (outside USA)	45 110
		Domestic (USA) graduate	336
		Outside European economic area	112
		International graduate (outside Canada)	123
		Primary Medical Qualification (PMQ) outside of the UK	136
	Graduation School	Without relatively "prestigious" training credentials (i.e., high-ranked medical school or residency program)	188
		From high outlier (Above the 90th percentile of the distribution of the percentage of graduates sued) medical school	212
	Medical Degree	Higher degree	47
		Doctors of osteopathic medicine (vs doctors of medicine)	274
	Graduation Time	Greater age at graduation	124
		Having passed Primary Medical Qualification (PMQ) more than 20 years ago	136
		A more recent medical school graduation year	337
	Previous Behaviours at School	Had a slightly lower mean undergraduate science GPA than did the control physicians, slightly lower MCAT scores, not to have passed at least one course on the first attempt in both the preclinical and clinical years of medical school, and displayed unprofessional behavior in medical school	111
		Failed the Medical Council of Canada Qualifying Examination (MCCQE) Parts I on their first attempt	123
		Worse professionalism ratings during residency, progressively lower scores on the ABIM certification examination, worse performance on the internal medicine certification examination	254

		Lower clinical skills examination (CSE) communication scores	261
		Difficulties in early/preclinical course, delayed graduation, poorer progress in the clinical course	306
		Lower undergraduate GPA, having concern/problem/extreme excerpts in their medical school file	335
	Trainee Status	All residents that left before graduation, and all categorical residents that left before completion of their chief clinical year(vs graduated residents, residents still in the program), non-categorical residents (vs categorical residents)	253
		Nonresidents (vs residents)	274
	Continuous Education	Respondents whose insurance providers did not require periodic educational courses	341
Competence or Health	Emotional Intelligence	Lower level of emotional intelligence	29
	Physician Impairment	Depressive symptoms	26
		Higher sleep-related impairment scores, lower professional fulfilment scores	30
		Higher burnout scores	30 31
		Having a General Health Questionnaire(GHQ) score (represents psychiatric morbidity) >4, higher scores in the Alcohol Use Disorders Identification Test	34
Professional Backgrounds	Medical Speciality	Calculi and oncology subspecialty (vs general urology)	196
		Cardiology	211 274
		Cardiothoracic surgery(vs cardiology)	211
		Dentists, dental prosthetists (vs oral health therapists, dental therapists and dental hygienists)	78
		Dermatology	123 148 284
		Emergency medicine	110 212 274 289
		Gastroenterology	72 211
		General practice	45 47 110 112 136 149 188 258 310
		Internal medicine	123
		Neuro-ophthalmology (vs comprehensive ophthalmology)	25
		Neurosurgery	114 274 353
		Non-internal medicine	48
		Nonophthalmic surgery, non-surgery (vs ophthalmology)	338
		Obsterics-gynaecology	34 45 110 123 148 149 150 197 212 274
		Ophthalmology (Ref=Internal medicine)	274
		Oral and maxillofacial surgery, orthodontics, periodontology and oral mucosa (vs other dental specialties)	49
		Otolaryngology	274 336
		Pediatrics	123
		Plastic surgery	148 274 284 349 353
		Psychiatry	45 76 110 149
		Radiology	212 274
		Urology (Ref=Internal medicine)	274
		Surgery	34 136 149 150 188 197 212 240 244 261 272 274
		Surgical oncology, medical oncology	337
		Specialist(without a GP specialty)	112 188 258

	Profession	Family practitioners	45 110 123
		Primary care practitioners	72 261
		Dental practitioners	78 149
		Psychologists (vs optometrists, physiotherapists, osteopaths and chiropractors)	76 149
		Chiropractors, osteopath (vs physiotherapists)	77 149
		Prosthetists	149
		Pharmacists	149
		GP proceduralists	255
	Practice Experience	Seniority fewer than 5 years	17
		In practice fewer than 20 years	61
		Longer in practice	36 46 110 123 124 126 153 241 272 285 341
		Qualified 31–40 years ago	112
		Greater career volume	36
	Employment Characteristics	No medical malpractice insurance	17
		One or more complaint or misconduct history	148 149 188 208 221 244 274 284 323
		Longer time since last complaint	284
		Not being board certified	45 61 110
		Having a teaching role	34 123
		Not having hospital privileges	123
		Not practising exclusively as a locum	123
		Primary employment being medical research or teaching	188
Work Practice	Operational Characteristics	Performing procedures requiring sedation/ anaesthesia	22 123
		Performing more split-thickness skin grafts	153
		Accepting new patients	123
		Using an electronic medical records system	123
		Not using an electronic medical records	126
		Using fewer resources	262
	Workload	Working longer hours	34 123 255
		Greater patient volume/ consultations/ clinical activity	125 139 208 221 241 244 285
		Higher workload (=number of patients/number of duties)	258
		No duty (vs groups with higher workload)	258
	Interaction with Patients	Influencing ("nudging") the patient to decline to screen	122
		Excluding patient from decision-making	122
		Spending less time with the patient	208
		Incorporating advertising into a higher volume refractive surgery practice	208
		Being more dominant and less concerned/anxious	320
		Informing patient about possible consequences if he or she refuses treatment	341
		Not using procedure-specific brochures	341
		Having worse rapport with patients	343
		Taking less time to explain	343
		Being not available	343

	Teamwork	Solo practice	123 126 188 310
		Non-solo practice	34
		No attendance at peer review meetings	34
		Comanagement (Preoperative and postoperative comanagement with optometrists)	208
System and Environmental Characteristics			
System Characteristics	Practice Setting	Aged-care departments(vs general wards)	12
		The departments of oral and maxillofacial surgery, periodontology and oral mucosa and the administration offices(vs the endodontic departments)	49
		Working in direct patient care (vs physicians employed in medical administration)	188
		Admission via emergency room (ref=outpatient department)	240
		Outpatient settings	347
	Practice Size	In small group practices of 2 to 4 people and 5 to 9 people (vs groups of 10 or more physicians)	126
	Type of Facility	General hospitals (vs specialised orthopedic hospitals)	8
		Public sector	12
		Academic/university	25 310 337 338
		For-profit chain facilities (vs non-profit, charitable and public facilities)	83
		Community pharmacy	257
	Organisation Characteristics	Hospitals with a large Medicaid patient population	150
Environmental Characteristics	Geographic Location	USA: New York	98
		USA: Wayne County	188
		USA:<br>Region I: Connecticut, Maine, Massachusetts, New Hampshire, Rhode Island, Vermont, non-US locations; Region II: Delaware, New Jersey, New York, Pennsylvania, Puerto Rico, Virgin Islands; Region III: Alabama, Arkansas, Florida, Georgia, Louisiana, Mississippi, North Carolina, South Carolina;Tennessee; Region IV: District of Columbia, Illinois, Indiana, Kentucky, Maryland, Michigan, Ohio, Virginia, West Virginia, Wisconsin.	221
		USA: west region	337
		Australia: metropolitan	77
		Australia: in regional and remote areas	149
		Australia: Queensland	283
		Australia: regional and remote region (vs Metropolitan)	353
		Denmark: the capital region	154
		Canada: Ontario (vs Quebec)	261
	Economic Environment	Lower-income patient populations	139
Issues of complaints or misconduct			
Issues of complaints or misconduct		Appointments, communications	20
		Diagnosis errors, procedural errors	72
		Substance use, honesty, a practitioner's mental health, sexual boundaries, and use and supply of medications	149
		Dissatisfaction with treatment, dissatisfaction with accessibility	336

Appendix D. Detailed findings on risk factors

ID #	Author & year	Title	Method	Risk Factors
8	Casali 2018[38]	Alleged malpractice in orthopaedics. Analysis of a series of medmal insurance claims	Chi-square test	• **Type of facility:** More than 95% of the claims concerned hospital-linked malpractice cases, with general hospitals accounting for 89% and specialized orthopedic hospitals accounting for only 11% ($p < 0.001$).
11	Otaki 2017[88]	Analysis of closed claims in the clinical management of rheumatoid arthritis in Japan	Fisher's exact test	• No significant difference
12	Taylor 2004[80]	Analysis of complaints lodged by patients attending Victorian hospitals, 1997-2001	Poisson regression	• **Type of facility:** Significantly more complaints ($P < 0.001$) were lodged by (or on behalf of) public patients (rate ratio, 2.1; 95% CI, 2.0–2.2).<br>• **Practice setting:** The complaint rate for general wards was 6.2/1000 patients (95% CI, 6.1–6.3). Intensive care units had a similar rate of 5.9/1000 (95% CI, 5.4–6.5), but aged-care departments had a significantly higher rate of 45.2/1000 (95% CI, 39.5–51.7), while emergency departments (1.9/1000; 95% CI, 1.8–2.0), operating theatres (1.0/1000; 95% CI, 1.0–1.1), day-procedure units (0.5/1000; 95% CI, 0.5–0.6) and outpatient departments (0.4/1000; 95% CI, 0.4–0.4) had significantly lower rates.
17	Lyu 2011[23]	Analysis of medical litigation among patients with medical disputes in cosmetic surgery in Taiwan	Multiple logistical regression	• **Practice experience, employment characteristics:** Surgeon factors seniority less than 5 years and no medical malpractice insurance or experience with medical litigation.
20	Sachdeo 2012[24]	An analysis of patient grievances in a dental school clinical environment	Chi-square test	• **Complaint types:** Complaints regarding appointments were prominent in all years. Conversely, the "other" category had very few complaints throughout the four years. By aggregating the data across years, the chi-square test for multiple categories indicated a statistically significant difference between the complaint types ($p<0.001$). When ignoring the other category and re-running the chi-square test for the four remaining complaint types, we found the difference was still significant ($p<0.001$). We found appointments to be the predominant category of complaint over the four years of the study, with communication as the second highest overall (Figure 2).
22	Kynes 2013[25]	An analysis of risk factors for patient complaints about ambulatory anesthesiology care	Regression model	• **Operational characteristics:** In the pediatric patient model, risk factors associated with the use of general anesthesia (versus not). In the adult patient model, risk factors associated with complaint risk included general anesthesia…
24	Tessler 2012[26]	Association between anesthesiologist age and litigation	Generalized estimating equation (GEE)	• **Age**: In univariate analysis with the less than 51 age group as the reference category, the litigation rate ratio for the 51–64 age group was 1.14 (95% CI: 0.99 – 1.32) and for the 65 and older age group was 1.50 (95% CI: 1.14 –1.97).Our analyses using disability weighted claims showed the 51–64 group to have 1.31 (95% CI: 0.95–1.80) and 65 and older group to have 1.94 (95% CI: 1.41–2.67) relative increase in disability compared to the less than 51 age group.

25	Fathy 2018[4]	Association between ophthalmologist age and unsolicited patient complaints	Multivariable Cox proportional hazards regression model	• **Age:** The 2 youngest age bands were associated with a statistically significant shorter time to first complaint. Compared with those aged 71 years or older, the risk of incurring a UPC for those aged 41 to 50 years was 1.73-fold higher (hazard ratio[HR], 1.73; 95%CI, 1.21-2.46; P = .002). Similarly, participants aged 31 to 40 years had a 2.36 times higher risk of incurring a UPC (HR, 2.36; 95% CI, 1.64-3.40; P < .001).<br>• **Medical speciality:** Only neuro-ophthalmology was associated with a statistically significant higher adjusted HR for time to first complaint when compared with comprehensive ophthalmology (HR, 1.97; 95% CI, 1.29-3.01; P = .002).<br>• **Type of facility:** Ophthalmologists at regional medical centers had a lower hazard rate for time to first complaint than ophthalmologists at academic medical centers (HR, 0.79; 95% CI, 0.65-0.97; P = .02).
26	Pereira-Lima 2019[39]	Association between physician depressive symptoms and medical errors: A systematic review and meta-analysis	Systematic Review and Meta-analysis	• **Physician impairment:** Similar to the results for the meta-analysis of physician depressive symptoms associated with subsequent medical errors, the meta-analysis of 4 longitudinal studies (involving 4462 individuals) found that medical errors associated with subsequent depressive symptoms had a pooled RR of 1.67 (95%CI, 1.48-1.87; $\chi 2$ = 1.85; P = .60; I2 = 0%; $\tau 2$ = 0), suggesting that the association between physician depressive symptoms and medical errors is bidirectional.
29	Shouhed 2019[40]	Association of emotional intelligence with malpractice claims: A review	Review	• **Emotional Intelligence:** This review suggests an indirect negative correlation between a physician's level of EI and his or her risk of litigation.
30	Welle 2020[41]	Association of occupational distress and sleep-related impairment in physicians with unsolicited patient complaints	Ordinal logistic regression models	• **Physician impairment:** Each 1-point increase in burnout and sleep-related impairment, on a 5-point scale, was associated with a 69% (odds ratio [OR], 1.69; 95% CI, 1.12-2.54) and 49% (OR, 1.49; 95% CI, 1.08-2.05) increased odds of being in the next higher PARS risk category, respectively, averaged across all 4 years.
31	Menon 2020[57]	Association of physician burnout with suicidal ideation and medical errors	Multivariate logistic regression models	• **Physician impairment:** In the adjusted model for self-reported medical errors, each SD-unit increase in burnout was associated with an increase in self-reported medical errors (OR, 1.48; 95%CI, 1.28-1.71).
34	Nash 2009[81]	Australian doctors' involvement in medicolegal matters: a cross-sectional self-report study	Univariate and multivariate logistic regression	• **Medical speciality:** Obstetricians/gynaecologists and surgeons had the highest risk of being involved in a current medicolegal matter. Obstetricians/gynaecologists and surgeons had the highest risk of being involved in a current medicolegal matter.<br>• **Gender, marital status, workload, physician impairment:** Other factors associated with higher risk of involvement in medicolegal matters were being male, being partnered or divorced/separated (rather than single), working longer hours, and having a General Health Questionnaire(GHQ) score (represents psychiatric morbidity) >4.<br>• **Location of education, teamwork, employment characteristics, physician impairment:** Factors that were significant in the univariate analysis but not in the multivariate model were the country in which the doctor's medical degree was

				obtained, attendance at peer review meetings, having a teaching role, type of practice (solo or nonsolo) and AUDIT score.
36	Dallal 2014[59]	Bariatric-related medical malpractice experience:survey results among ASMBS members	Logistic multivariable regression model	• **Practice experience:** The number of years in practice (OR 1.03; P = .03) and greater career volume (OR 8.5; P = .01) were independently associated with an increased likelihood of having a bariatric related medical malpractice claim at some point in their careers.
45	Kohatsu 2004[60]	Characteristics associated with physician discipline: a case-control study	χ2, and t tests, multivariable logistic regression analyses	• **Age, gender, employment characteristics, medical speciality, profession, location of education:** In univariate analysis, cases were more likely than controls to be male; not board certified; in the specialties of family practice, general practice, obstetrics and gynecology, or psychiatry; and an international medical school graduate. Increasing age in 20-year intervals and male sex were positively and independently associated with an increased likelihood of discipline. Board certification was associated with a significantly reduced risk of discipline. Relative to specialists in internal medicine, specialists in radiology and pediatrics were at significantly lower risk of discipline, while specialists in family practice, general practice, obstetrics and gynecology, and psychiatry were at significantly higher risk. International medical education was associated with a significantly elevated risk of discipline.
46	Birkeland 2013[27]	Characteristics of complaints resulting in disciplinary actions against Danish GPs	Multiple logistic regression model	• **Practice experience:** With regard to patient and general practitioner characteristics, higher general practitioner professional seniority was associated with increased odds of discipline (OR=1.97 per 20 additional years of professional seniority, p=0.01).
47	Cunningham 2003[93]	The characteristics of doctors receiving medical complaints: a cross-sectional survey of doctors in New Zealand	Chi-square test	• **Gender:** Male doctors were more likely to have received a complaint (p <0.01).<br>• **Medical speciality:** Vocationally registered general practitioners received more complaints than either hospital-based specialists or doctors on the general register (p <0.01).<br>• **Medical degree:** More doctors holding higher degrees received complaints (p <0.01)
48	Liu 2015[84]	Characteristics of internal medicine physicians disciplined by professional colleges in Canada	Significant test	• **Medical speciality:** A rate difference over the cumulative 14-year study period demonstrated that Internal Medicine physicians incur 5.18 fewer disciplinary cases than non-Internal Medicine physicians per 10,000 physician years (95% CI 3.62–6.73; P<0.001)
49	Liu 2018[42]	Characteristics of medical disputes arising from dental practice in Guangzhou, China: An observational study	χ2 test, binary logistic regression analysis	• **Medical speciality, practice setting:** Compared with the endodontic department, the departments of oral and maxillofacial surgery (OR 3.05, 95% CI 1.33 to 7.01), periodontology and oral mucosa (OR 3.19, 95% CI 1.36 to 7.47) and the administration office (OR 3.56, 95% CI 1.38 to 9.19) were more likely to have physical conflicts.
61	Clay 2003[61]	Characteristics of physicians disciplined by the State Medical Board of Ohio	Multivariate logistic regression models	• **Employment characteristics, practice experience:** Disciplined physicians were significantly less likely to be board certified (OR, 0.65; CI, 0.46-0.92) and

				significantly more likely to be in practice fewer than 20 years (OR, 1.51; 95% CI, 1.08-2.13).
72	Panuganti 2020[43]	Colorectal cancer litigation: 1988-2018	Univariate analysis, χ2, and t tests	• **Complaint types:** Plaintiffs overwhelmingly alleged errors in diagnosis (n=192, 80%) over procedural errors (n=40, 17%) as the major reason for litigation (P< 0.01).<br>• **Profession, medical speciality:** Accordingly, the primary defendants in most lawsuits were primary care practitioners (n= 61, 25.4%) and gastroenterologists (n = 55, 22.9%).<br>• **Gender:** Among medical providers listed as primary defendants (n = 170, 71%), most were identified as men (n = 147, 86%) than women (n= 23, 14%) (P <0.01).
76	Veness 2019[44]	Complaint risk among mental health practitioners compared with physical health practitioners: A retrospective cohort study of complaints to health regulators in Australia	χ2 tests, multivariate negative binomial regression analysis	• **Medical speciality:** The complaint rate among psychiatrists was more than double than among physicians (119.1 vs 48.0 complaints per 1000 practice years, p<0.001).<br>• **Profession:** The complaint rate among psychologists was nearly treble than among other allied health practitioners (21.9 vs 7.5 complaints per 1000 practice years, p<0.001).<br>• **Age:** Older mental health practitioners had a higher risk of complaints than their younger peers, after adjusting for sex and practice location. For both psychiatrists and psychologists, complaint risk increased steadily by age band, with practitioners aged≥ 65 years having around twice the risk of complaint compared with those aged 36–45 years.(psychiatrists: IRR 2.37, 95% CI 1.95 to 2.89; psychologists: IRR 1.78, 95% CI 1.47 to 2.14).<br>• **Gender:** Male psychiatrists and psychologists had higher complaint rates than their female peers (psychiatrists: IRR 1.61, 95% CI 1.39 to 1.85; psychologists: IRR 1.85, 95% CI 1.65 to 2.07).
77	Ryan 2018[2]	Complaints about chiropractors, osteopaths, and physiotherapists: a retrospective cohort study of health, performance, and conduct concerns	Chi-square test, multivariate negative binomial regression model	• **Profession:** The complaint rate for chiropractors was three times higher than for osteopaths and six times higher than for physiotherapists (29 vs. 10 vs. 5 complaints per 1000 practice years, p < 0.001). Chiropractors had a higher complaint rate compared to physiotherapists (IRR = 4.45, 95% CI = 3.83-5.16), after adjusting for age, sex, practice location, practice years as well as state/territory.<br>• **Age:** Across all three professions, practitioners aged 66 years or older had a higher rate of complaints compared to those aged 35 years and younger (IRR = 2.28, 95% CI = 1.62 – 3.21).<br>• **Gender:** Male practitioners had 2.4 times the rate of being the subject of a complaint compared with their female peers (IRR = 2.43, 95% CI = 2.10 – 2.82).<br>• **Geographic location:** Practice location was a weaker predictor; those who practiced in metropolitan areas had 1.2 times the rate compared with those who practiced in rural and remote areas (IRR = 1.23, 95% CI = 1.02 – 1.48).

78	Thomas 2018[45]	Complaints about dental practitioners: an analysis of 6 years of complaints about dentists, dental prosthetists, oral health therapists, dental therapists and dental hygienists in Australia	Incidence rate ratios (IRRs), negative binomial regression analysis	• **Profession:** After adjusting for age, sex, practice location and jurisdiction, the overall complaint rate for dental practitioners was 42.7 per 1000 practitioners per year (95% CI 41.0–44.4) – higher than for any other health profession.<br>• **Medical speciality:** Within the dental profession, dentists had the highest rate of complaint (56.9 per 1000 practitioners per year, 95% CI 54.6–59.3), followed by dental prosthetists (50.0 per 1000 practitioners per year, 95% CI 42.8–57.2). Allied dental professionals (oral health therapists, dental therapists and dental hygienists) had significantly lower rates of complaint (11.2 per 1000 practitioners per year, 95% CI 9.1–13.3). After adjusting for age, sex, remoteness, and jurisdiction, dentists and dental prosthetists had five times higher risk of complaints compared with allied dental professionals (dentists IRR = 5.1; 95% CI 4.2–6.2; dental prosthetists IRR = 4.5; 95% CI 3.5–5.7).<br>• **Age:** When compared with dental practitioners aged 35 years or younger, older practitioners had higher risks of receiving a complaint (e.g. IRR = 1.6 for 36–45 age group, IRR = 1.6 for 46–55, IRR = 1.5 for 56–65 and IRR = 1.8 for ≥66).<br>• **Gender:** The rates for male practitioners were 50% higher than for female practitioners (IRR = 1.5; 95% CI 1.4–1.6).
83	McGregor 2011[28]	Complaints in for-profit, non-profit and public nursing homes in two Canadian provinces	Significant test	• **Type of facility:** Compared with for-profit chain facilities, non-profit, charitable and public facilities had significantly lower rates of complaints in Ontario. Likewise, in British Columbia's Fraser Health region, non-profit owned facilities had significantly lower rates of complaints compared with for-profit owned facilities.
98	Baker 2013[29]	The demography of medical malpractice suits against radiologists	Poisson regression	• **Geographic location:** Adjusted for age and state, radiologists in Alabama had the lowest rate of malpractice suits per 100 practice-years for men (0.95; 95% confidence interval [CI]; 0.73, 1.28) and women (0.70; 95% CI: 0.52, 0.96) compared with those in New York, who had the highest rate of suits for men (5.65; 95% CI: 5.09, 6.26) and women (4.13; 95% CI: 3.54, 4.80).<br>• **Gender:** Overall, male radiologists had a higher rate of being sued than did their female counterparts (IRR = 1.37; 95% CI: 1.20, 1.56).
110	Khaliq 2005[62]	Disciplinary action against physicians: Who is likely to get disciplined?	Kaplan-Meier analysis, univariate analysis, multivariate Cox proportional hazards regression model	• **Practice experience:** Kaplan-Meier analysis revealed that rate of disciplinary action over time increased with each successive 10-year interval since licensure. The estimates of unadjusted rate of disciplinary action were found to be 1.3%(95% confidence interval[CI], 1.1-1.5) within 10 years since licensure, 2.8%(95% CI, 2.5-3.2) within 20 years, 4.3%(95%CI, 3.8-4.9) within 30 years, 6.6%(95% CI, 5.8-7.5) within 40 years, 8.6%(95% CI, 7.3-10.1) within 50 years, and 11.0%(95% CI, 8.5-14.1) within 60 years.<br>• **Gender, ethnicity, employment characteristics, location of education, medical speciality, profession:** Univariate Cox proportional hazards model showed that factors associated with an increased risk of disciplinary action included being a man, non-white, non-board-certified, and a foreign medical graduate, and belonging to the specialties of family medicine, general practice, psychiatry, obsteric-gynecology, and emergency medicine. The multivariate Cox proportional hazards model also showed risk factors for disciplinary action to include being a man, non-white, non-board-certified, and belonging to the specialties of family medicine,

				general practice, psychiatry, obsteric-gynecology, and emergency medicine. However, foreign medical graduate status was no longer associated with a significantly higher risk, and internal medicine and cardiology were no longer associated with a significantly lower risk of disciplinary action. Radiology was still associated with a lower relative risk of disciplinary action.
111	Papadakis 2005[63]	Disciplinary action by medical boards and prior behavior in medical school	Chi-square test, conditional logistic-regression models	• **Previous behaviour at school:** The disciplined physicians had a slightly lower mean undergraduate science GPA than did the control physicians. MCAT scores were also slightly lower among the disciplined physicians, as were NBME Part I scores and USMLE Step 1 scores. Disciplined physicians were roughly twice as likely as control physicians not to have passed at least one course on the first attempt in both the preclinical and clinical years of medical school. Unprofessional behavior was associated with an increase, by a factor of three, in the risk of subsequent disciplinary action, and it accounted for the largest population attributable risk (26 percent). Low MCAT scores and low grades in the first two years of medical school were also significant predictors, with a population attributable risk of disciplinary action of 1 percent and 7 percent, respectively.
112	Unwin 2014[89]	Disciplined doctors: Does the sex of a doctor matter? A cross-sectional study examining the association between a doctor's sex and receiving sanctions against their medical registration	Mantel-Haenszel analyses and tests, logistic regression model	• **Gender:** There was a higher proportion of male doctors who had sanctions against their registration when compared with female doctors (1.1% of all male doctors compared with 0.4% of all female doctors, $\chi2=505.4$, $p<0.001$). After taking into account the number of years since PMQ and world region where the doctor received their PMQ and specialty, female doctors had nearly a third of the odds of having sanctions on their registration compared with male doctors (OR: 0.37, 95% CI: 0.33 to 0.41, $p<0.0001$).<br>• **Practice experience, location of education, medical speciality:** There was strong evidence for an association between receiving sanctions and the number of years since receipt of PMQ, with doctors who qualified 31–40 years ago having the highest proportion of sanctions; world region of PMQ, with doctors who qualified outside the EEA with the highest proportion of doctors with sanctions; and specialty, with doctors on both the Specialist and GP Registers having the highest proportion of doctors with sanctions.
114	Dambrino 2021[9]	Do neurosurgeons receive more patient complaints than other physicians? Describing who is most at risk and how we can improve	Pearson chi-square test; Kruskal-Wallis test	• **Medical speciality:** Neurosurgeons had more average total UPCs per physician (8.68; 95% CI 7.68–9.67) than nonsurgeons (3.40; 95% CI 3.33–3.47) and other surgeons (5.01; 95% CI 4.85–5.17; $p < 0.001$).
122	Birkeland 2021[46]	Does greater patient involvement in healthcare decision-making affect malpractice complaints?A large case vignette survey	Linear regression	• **Interaction with patients:** Compared with scenarios that involved shared decision-making (SDM), neutral information, or nudging in favor of screening, the urge to complain increased if the patient was excluded from decision-making or if the doctor had nudged the patient to decline screening (mean Likert differences .12 to .16, $p < .001$). With neutral involvement or nudging in favor of intervention, the desire to complain depended highly on the decision reached and on the patient's course. This dependence was smaller with SDM.
123	DeChamplain 2020[47]	Does pass/fail on medical licensing exams predict future physician performance in	Separate multivariate quasi-Poisson regression models	• **Previous behaviour at school:** Candidates who failed the MCCQE I on their first attempt had 27% more complaints lodged against them, compared to those who passed.

		practice? A longitudinal cohort study of Alberta physicians		• All 13 CPSA registration variables were significantly associated with a higher frequency of patient complaints. Specifically, a larger number of complaints was associated with: (1) **Practice experience:** A higher number of years since initial registration (7% more complaints per year) (2) **Profession:** Family Medicine (over double the number of complaints compared to OS physicians) (3) **Workload:** More days of providing medical services per week (26% more complaints per day) (4) **Location of education:** International medical graduates (44% more complaints than Canadian medical graduates) (5) **Operational characteristics:** (33% more complaints than physicians not accepting new patients) (6) **Gender:** Male physicians (33% more complaints than female physicians) (7) **Age:** Older physicians (2% more complaints per year of age) (8) **Operational characteristics:** Performing procedures requiring sedation/ anaesthesia (27% more complaints for those physicians performing procedures requiring sedation/anaesthesia) (9) **Operational characteristics:** (23% more complaints for those physicians using an EMR) (10) **Employment characteristics:** Not having hospital privileges (22% more complaints for those physicians not having privileges) (11) **Employment characteristics:** Not practicing exclusively as a locum (66% more complaints for those physicians not practicing exclusively as a locum) (12) **Employment characteristics:** Non-clinical teaching (1% more complaints for those physicians that teach with no provision of medical services) (13) **Medical speciality:** Primary specialty (included internal medicine, dermatology, pediatrics and obstetrics/gynecology.) (21% fewer complaints than other specialty physicians) (14) **Teamwork:** Not practicing in a group (15% more complaints for those physicians who did not practice in a group, e.g., solo practitioners)
124	Birkeland 2018[48]	Education trajectories and malpractice complaints—A study among Danish general practitioners	Multiple logistic regression model	• **Graduation time:** Greater age at graduation was associated with increased odds of later complaints, but decreased odds of complaints leading to critique by a disciplinary board.<br>• **Practice experience:** In addition, the time following specialisation, in particular, was associated with increased odds of complaints.
125	Schaffer 2020[49]	The effect of clinical volume on annual and per-patient encounter medical malpractice claims risk	Linear regression	• **Workload:** As clinical volume increased, the percent of physicians with a malpractice claim increased linearly. Among all physicians studied, for each decile increase in clinical volume, there was a 0.373% increase in physicians with a malpractice claim (95% confidence interval, 0.301%-0.446%; P < 0.0001).
126	Virapongse 2008[64]	Electronic health records and malpractice claims in office practice	Pearson χ2 test, the Wilcoxon rank sum test, and the unpaired, 2-tailed t test, logistic regression analysis	• **Gender:** Paid malpractice claims were more common among male physicians (11.1%) than female physicians (5.6%) (P=.003).<br>• **Practice experience:** Paid malpractice claims were more common among physicians who had been in practice longer. For example, 15.2% of physicians who graduated from medical school more than 20 years ago had paid malpractice claims in the past 10 years compared with 5.8% of physicians who had graduated within the past 20 years (P＜.001).<br>• **Teamwork, practice size:** Practice size was also correlated with malpractice claims. Paid malpractice claims were more common among physicians in solo practice (43.7%) and among those in small group practices of 2 to 4 people

				(29.1%) and 5 to 9 people (19.4%) than among physicians who practiced in groups of 10 or more physicians (7.8%).<br>• **Operational characteristics:** In logistic regression analysis, controlling for physician sex, year of medical school graduation, and practice size, a significant association was found, indicating that physicians with EHRs were less likely to have paid malpractice claims (adjusted OR, 0.19; 95% CI, 0.05-0.78).
136	Croft 2019[11]	A focused mapping review and synthesis of a priori risk factors associated with medical misconduct	A focused mapping review and synthesis	• Through the identification of four a priori risk factors, we developed a model of risk associated with medical misconduct: (1) **Gender:** being male, (2) **Location of education:** primary medical qualification (PMQ) outside of the UK, (3) **Medical speciality**: working within general practice and surgical specialties, and (4) **Graduation time:** having passed PMQ more than 20 years ago.
139	Birkeland 2019[50]	General practice location and malpractice litigation	Multivariate logistic regression	• **Workload:** Larger patient list size was associated with higher rates of malpractice litigation (odds ratio (OR) 1.05 per 100 patients).<br>• **Economic environment:** Litigation was less frequent in settings with higher income patient populations (OR 0.65), although where it did occur the criticism seemed much more likely to be justified (OR 6.03).
148	Bismark 2013[7]	Identification of doctors at risk of recurrent complaints: A national study of healthcare complaints in Australia	Multivariable regression analysis	• **Employment characteristics:** Compared with doctors with one prior complaint, doctors with two complaints had nearly double the risk of recurrence (HR 1.93; 95% CI 1.79 to 2.09), and doctors with five prior complaints had six times the risk of recurrence (HR 6.16; 95% CI 5.09 to 7.46). Doctors with 10 or more prior complaints had 30 times the risk of recurrence (HR 29.56; 95% CI 19.24 to 45.41).<br>• **Medical speciality:** Risk of recurrence also varied significantly by specialty. Compared with general practitioners, plastic surgeons had twice the risk (HR 2.04; 95% CI 1.75 to 2.38), and risks were approximately 50% higher among dermatologists (HR 1.56; 95% CI 1.30 to 1.88) and obstetrician-gynecologists (HR 1.50; 95% CI 1.29 to 1.76).<br>• **Gender:** Male doctors had a 40% higher risk of recurrence than their female colleagues (HR 1.36; 95% CI 1.23 to 1.50).<br>• **Age:** Compared with doctors 35 years of age or younger, older doctors had 30–40% higher risks of recurrence.
149	Spittal 2019[1]	Identification of practitioners at high risk of complaints to health profession regulators	Recurrent-event survival analysis	• **Gender:** Male practitioners' complaint risk was 1.5 times that of female practitioners.<br>• **Age:** There was no significant difference in complaint risks for practitioners 26–35 years and those aged ≤25 years, but practitioners in the older age groups had 1.5 to 2.1 times higher risk and it generally increased with age.<br>• **Geographic location：** Compared to practitioners working in major cities, those based in regional Australia had 1·1 times higher complaint risk and those in remote areas had 1·3 times the risk.<br>• **Profession, medical speciality:** Risks varied widely by profession and medical specialty. Compared to medical radiation practitioners (the profession with the

				lowest risk and the reference group), the risk of a complaint was substantially higher for all the medical specialties, especially obstetrics and gynaecology (HR = 16.2), psychiatry (HR = 16.1), surgery (HR = 13.3) and general practice (HR = 11.2). Dentists and dental prosthetists were also at very high risk (HR = 11.5). Chiropractors (HR = 6.5), psychologists (HR = 6.0), and pharmacists (HR = 5.6) had elevated risks of complaints, whereas the risks for enrolled nurses (HR = 1.8), registered nurses (HR = 1.8), physiotherapists (HR = 1.6), occupational therapists (HR = 1.5) and midwives (HR = 1.0), were relatively close to those of the reference group.<br>• **Employment characteristics:** Complaint risk increased monotonically with the number of prior complaints. Compared to those with no prior complaints, practitioners with 1 prior complaint had 2·6 times higher risk of accruing another complaint; those with 3 prior complaints had 5.1 times the risk and those with ≥7 prior complaints had 12.0 times the risk.<br>• **Complaint types:** The highest risks of additional complaints followed complaints relating to concerns about substance use (HR = 4.2), honesty (HR = 3.6), a practitioner’s mental health (HR = 3.3), sexual boundaries (HR = 2.4) and use and supply of medications (HR = 2.2).
150	Rolph 2007[65]	Identifying malpractice-prone physicians	Empirical Bayes methods in a negative Binomial Regression	• **Medical speciality:** As expected, specialty group is important, with major surgery and obstetrics having the highest claims rates.<br>• **Gender, age:** As expected, doctors who are male and who are older have higher claims rates. (We capped the age “effect” at 50 since the risk plateaus there.)<br>• **Organizational factors:** Being on staff of a hospital with a large Medicaid patient population is associated with having a higher claims rate, while being on staff at a large inner-city hospital or at a hospital with more than one ICU is associated with lower malpractice claims rates.
153	Perlis 2006[66]	Incidence of and risk factors for medical malpractice lawsuits among Mohs surgeons	Chi-square test, Student’s t-test, Mann-Whitney test	• **Practice experience:** The longer a physician reported practicing Mohs surgery, the higher the likelihood of having been sued (2 [4df] = 23.3923, p < .001).<br>• **Operational characteristics:** Physicians who performed more splitthickness skin grafts were more likely to be sued (split t = 2.32, p = .02).
154	Nikoghosyan-Bossen 2012[30]	Increased number of ear-nose-throat malpractice complaints in Denmark	Chi-square test	• **Geographic location:** Table 1 shows a significant difference in the number of complaints between regions with the Capital region having the highest rate (χ2 = 106, degree of freedom (df) = 4, p < 0.000).
188	Weycker 2000[68]	Medical malpractice among physicians: who will be sued and who will pay?	Multivariate logit models(logistics regression)	• **Graduation school:** Physicians with relatively “prestigious” training credentials (i.e., high ranked medical school or residency program) were less likely to incur an adverse malpractice record.<br>• **Gender:** Male physicians were 1.393–1.798 times as likely than female physicians to incur an adverse malpractice record in 1985–1989.<br>• **Practice setting:** As expected, physicians working in direct patient care were significantly more likely (odds of 1.708–2.505) to incur an adverse record in 1985–

				1989 relative to physicians employed in medical administration (the reference group).<br>• **Employment characteristics:** The odds of developing an “adverse record” were significantly greater for physicians with any level of exposure to malpractice in the 1980–1984 period, relative to physicians with no claims history (the reference group). Physicians whose primary employment was medical research or teaching were more likely to incur some claims (odds ratio of 1.296) and more likely to incur large claims (odds ratio of 1.146).<br>• **Teamwork:** Solo practitioners had slightly higher odds of an adverse record relative to physicians in a group practice, although two estimates were statistically significant.<br>• **Geographic location:** The odds of an adverse record in the outcome period also were significantly higher for Wayne County physicians, where claim rates and malpractice awards have historically been high. These physicians had odds of 1.187–1.397 times that of physicians practicing elsewhere in the state.<br>• **Medical speciality:** Since specialty groups were categorized by level of risk, we expected physicians in the highest risk group, surgical specialty, to have the highest odds, followed by general practitioners, medical specialists, and the “other” specialty group.
196	Stimson 2010[58]	Medical malpractice claims risk in Urology: An empirical analysis of patient complaint data	Chi-square test	• **Medical speciality:** Subspecialty and distribution of risk scores were significantly associated(p＜0.001). Specifically, of the subspecialties accounting for more than 5% of the cohort, calculi and oncology subspecialists had higher mean risk scores (20.5 and 19.5, respectively; median 2.0 and 8.0, respectively) compared with general urologists (mean 5.7, median 0.0).
197	Wu 2009[90]	Medical malpractice experience of Taiwan: 2005 versus 1991	Logistic regression	• In the 1991 survey: **Gender:** Female physicians had significantly lower odds of malpractice (odds ratio, OR = 0.53, P = 0.043). **Age:** Compared with physicians who were 25–34 years of age, physicians 35–44, 45–54, 55–64 and 65 years of age or older, had significantly higher risk of experiencing malpractice events (OR = 1.98, P = 0.001; OR = 2.66, P < 0.001; OR = 18.4, P = 0.012; OR = 1.86, P = 0.008, respectively). Malpractice experiences were more frequent in physicians of surgery or obstetrics/gynaecology specialties. **Medical speciality:** Compared with general physicians, obstetricians/gynaecologists had the highest risk of malpractice (OR = 1.76, P = 0.003), followed by surgeons (OR = 1.62, P = 0.011).<br>• In the 2005 survey: **Gender:** Female physicians did not have a significantly lower risk of malpractice in 2005. **Age:** Compared with physicians in the youngest age group, physicians 45–54 and 55–64 years of age had a significantly higher risk of experiencing malpractice (OR = 2.27, P = 0.001; OR = 2.46, P = 0.002, respectively). **Medical speciality:** Surgeons and obstetrician/gynaecologists still had a significantly higher risk of malpractice (OR = 1.85, P = 0.045; OR = 2.40, P = 0.004, respectively). In 2005, 56.5% surgeons and 73.2% obstetrician/gynaecologists had some sort of malpractice experience.

208	Abbott 2003[67]	Medical malpractice predictors and risk factors for ophthalmologists performing LASIK and photorefractive keratectomy surgery	Logistic regression analysis	• **Workload:** The most important predictor for a physician incurring a claim or lawsuit was patient volume, with greater probability of a claim among those treating more patients (OR =31.4 for ＞1000 procedures/year vs. 5–20 procedures/year, 95% CI=7.9–125, P= 0.0001).<br>• **Gender:** Using univariate analysis and without controlling for volume, the OR for a female physician incurring a claim or lawsuit relative to a male was 0.24 (95% CI= 0.06–0.99, P = 0.048).<br>• **Interaction with patients:** The incorporation of advertising into a higher volume refractive surgery practice showed a significant positive correlation with those who were involved in a claim or lawsuit. The data showed that spending more time with the patient lowered the risk of incurring a claim or lawsuit (P=0.003) and became increasingly significant with higher volume surgeons (P =0.0001).<br>• **Teamwork:** In examining the results from the practice pattern survey using multivariate analysis, surgeons performing over 100 cases per year and who comanaged demonstrated a significantly higher OR of incurring a claim or lawsuit (13.90, 95% CI=4.48–43.10, P=0.0001).<br>• **Employment characteristics:** Physicians having one or more prior claims or lawsuits was the only other predictor examined that remained statistically significant when controlled for patient volume(OR=6.4, 95% CI =2.5–16.4, P =0.0001).
211	Mangalmurti 2014[69]	Medical professional liability risk among US cardiologists	Significant test	• **Medical speciality:** Cardiologists faced slightly higher annual rates of MPL claims and indemnity payments compared to physicians as a whole and general internists, but lower rates than gastroenterologists and cardiothoracic surgeons. For example, the percent of cardiologists facing a claim in a given year was 8.6%, compared to 6.6% among general internists (p <0.001), 11.6% among gastroenterologists (p < 0.001), and 18.9% among cardiothoracic surgeons (p<0.001).
212	Waters 2003[70]	Medical school attended as a predictor of medical malpractice claims	Logistic regression	• **Age:** Increasing age is positively associated with the probability of malpractice claims experience, but at a decreasing rate.<br>• **Medical speciality:** Obstetricians/gynecologists, surgical specialists, general surgeons, radiologists, and emergency medicine specialists were more likely to be sued than the comparison category.<br>• **Graduation school:** At the other end of the spectrum, physicians from high outlier medical schools were more likely to have a claim against them.
221	Guidera 2012[31]	Midwives and liability: Results from the 2009 nationwide survey of certified nurse-midwives and certified midwives in the United States	Chi-square tests, t test, Mann-Whitney U test, multiple logistic regression model	• **Age:** There was a significant difference in age between midwives who had and had not been involved in a lawsuit (P ＜.0001).<br>• **Employment characteristics:** The mean number of estimated births attended in their careers by midwives who had been involved in lawsuits was nearly double that of midwives who had not been involved in litigation (1650 vs 885; P ＜ .0001).<br>• **Workload:** for each additional birth attended, the odds of being involved in a lawsuit increase by 5.4% (P ＜ .001).

				• **Geographic location:** Region VI, chosen on the basis that this region represented central tendencies for being involved in litigation. The CNMs/CMs practicing in Region II (the Northeast, Puerto Rico, and the Virgin Islands) have 1.69 times the risk of being involved in litigation than those in Region VI (P = .017). Conversely, the odds of being in litigation for CNMs/CMs practicing in Region V (the Southwest and Midwest) are 0.581 that of CNMs/CMs practicing in Region VI (P = .018).
240	Wu 2009[91]	Patient characteristics predict occurrence and outcome of complaints against physicians: A study from a medical center in central Taiwan	Logistic regression analysis	• **Practice setting:** RMF cases were associated with… admission via emergency room (OR = 1.59, p = 0.005), admission to surgical specialty (OR = 1.89, p = 0.004).<br>• **Medical speciality:** From multivariate analysis, we found that only admission via the ER (adjusted OR=1.62, p=0.005), admission to surgical specialty (adjusted OR=1.86, p = 0.001), and living in an urban area (adjusted OR=1.93, p<0.001) were independent risk factors for filing an RMF case.
241	Birkeland 2013[32]	Patient complaint cases in primary health care: What are the characteristics of general practitioners involved?	Logistic regression	• **Practice experience:** With regard to complaints concerning daytime services (n = 265), the professional seniority of the general practitioner was positively associated with the odds of receiving a complaint decision (OR = 1.44 per 20 years of seniority; CI 95%, 1.04–1.98).<br>• **Workload:** Likewise, having more consultations per day was associated with increased odds (OR = 1.29 per 10 extra consultations per day; CI 95%, 1.07–1.54).
244	Hickson 2002[71]	Patient complaints and malpractice risk	χ2 analysis, Logistic regression	• **Medical speciality, workload:** High complaint generation, surgical specialty, and higher levels of clinical activity were all significantly associated with each measure of risk management activity.<br>• **Employment characteristics:** Surgeons named in a single lawsuit generated significantly more complaints than surgeons with no lawsuits(mean complaints=16.7 vs 6.1; P＜0.001)<br>• **Gender:** Female physicians were less likely than male physicians to be involved with RMFs.
253	Resnick 2006[72]	Patterns and predictions of resident misbehavior-A 10-year retrospective look	T-test	• **Trainee status:** Both the entire group of residents that left before graduation and all categorical residents that left before completion of their chief clinical year had statistically significant more complaints on an annual basis (0.65, 0.41) when compared with all residents in the study and those who completed the program(0.15, 0.07) with p＜ 0.05.. When normalized for time spent at the University of Pennsylvania, categorical residents received substantially fewer complaints on an annual basis than did their undesignated preliminary counterparts.
254	Papadakis 2008[73]	Performance during internal medicine residency training and subsequent disciplinary action by state licensing boards	Chi-square tests, Cochran–Armitage trend tests, t tests, Kendall–Tau tests, Multivariable Cox proportional hazards regression model	• **Previous behaviour at school:** Unadjusted analyses of diplomates showed that progressively better professionalism ratings during residency were associated with progressively less risk for subsequent disciplinary actions. Unadjusted analyses showed that progressively higher scores on the ABIM certification examination were associated with decreasing risks for subsequent disciplinary actions. Multivariate Cox proportional hazard analysis demonstrated that a low

				professionalism rating (＜4) independently predicted disciplinary action (hazard ratio, 1.7 [CI, 1.3 to 2.2]). Multivariate Cox proportional hazard analysis revealed that better performance on the internal medicine certification examination independently reduced the likelihood for disciplinary action (hazard ratio, 0.7 [CI, 0.6 to 0.7] for American or Canadian medical school graduates and 0.9 [CI, 08 to 1.0] for international graduates)
255	Nash 2009[82]	Personality, gender and medico-legal matters in medical practice	χ2 tests, t tests	• **Profession:** A higher proportion of proceduralists experienced a medico-legal matter (69.3%) than non-proceduralists (53.9%) (x2=11.78, df=1, p＜0.001).<br>• **Gender:** A significantly higher proportion of males (65.7%) than females (46.5%) reported experiencing a medicolegal matter (x2=19.44, df=1, p＜0.001).<br>• **Workload:** There was also a significant interaction between hours worked per week and number of current/past medico-legal matters on the combined personality factors(Wilks'λ=0.92; $F_{12,810}$=2.06, p=0.017).
257	Phipps 2011[33]	Pharmacists subjected to disciplinary action: characteristics and risk factors	Univariate log-linear analysis, multivariate logistic regression	• **Type of facility:** According to the Wald criterion, only the pharmacist's sector had a statistically significant effect on the classification. Specifically, pharmacists working in community pharmacy were at increased risk of being disciplined when compared to pharmacists in non-patient-facing roles.
258	Bratland 2020[12]	Physician factors associated with increased risk for complaints in primary care emergency services: a case -control study	Multivariable logistic regression	• **Medical speciality:** Adjusted for the other variables, physicians without GP specialty had a higher (double) risk of a complaint compared to those with a GP specialty the odds ratio (OR) was 2.05, 95% confidence interval (CI): [1.10–3.81].<br>• **Workload:** Compared to this group with no duty, the adjusted OR were significantly lower for evoking a complaint in the low, medium low and medium high workload. This group had the highest odds ratio for a complaint compared to groups with higher workload. The adjusted OR were significantly lower for evoking a complaint in the low, medium low and medium high workload, compared to no duty in the fourteen day period.
261	Tamblyn 2007[85]	Physician scores on a national clinical skills examination as predictors of complaints to medical regulatory authorities	Multivariate Poisson regression models	• **Previous behaviour at school:** Lower clinical skills examination (CSE) communication scores were associated with a higher rate of retained complaints, particularly in the lowest quartile of these scores.<br>• **Gender, medical speciality, profession, geographic location:** In multivariate models that adjusted for other physician characteristics, significantly higher complaint rates also were found for male vs female physicians, surgeons and primary care physicians vs medical subspecialists, and physicians practicing in Ontario vs those practicing in Quebec.
262	Jena 2015[74]	Physician spending and subsequent risk of malpractice claims: Observational study	Linear regression models	• **Operational characteristics:** In analyses that relied on variation in risk adjusted spending within the same physician over time (that is, within physician analysis), greater physician spending in a given year continued to be negatively correlated with the probability of facing an alleged malpractice incident in the subsequent year, across specialties.

272	Bismark 2011[34]	Prevalence and characteristics of complaint-prone doctors in private practice in Victoria	Univariate and multivariate logistic regression	• **Gender, medical speciality, location of education, practice experience:** Univariate analyses showed that complaint prone doctors were more likely than control doctors to be male, surgeons or psychiatrists, have trained in Australia, and have been in practice for at least 30 years. In multivariate analysis, surgeons had ninefold greater odds of being complaint prone than GPs (odds ratio [OR], 8.90; 95% CI, 3.69–21.50) and psychiatrists had more than fourfold greater odds of being complaint-prone (OR, 4.59; 95% CI, 1.46–14.43). Doctors trained overseas had lower odds of being complaint-prone than Australian-trained doctors (OR, 0.31; 95% CI, 0.13–0.72).
274	Studdert 2016[8]	Prevalence and characteristics of physicians prone to malpractice claims	Multivariable survival analysis, Anderson–Gill model	• **Employment characteristics:** In multivariable analysis, physicians' risk of future paid claims increased monotonically with their number of previous paid claims.<br>• **Medical speciality:** As compared with the risk of recurrence among internal medicine physicians, the risk of recurrence was approximately double among neurosurgeons, orthopedic surgeons, general surgeons, plastic surgeons, and obstetrician–gynecologists.<br>• **Gender:** Male physicians had a 38% higher risk of recurrence than female physicians.<br>• **Age:** The risk of recurrence among physicians younger than 35 years of age was approximately one third the risk among their older colleagues.<br>• **Trainee status, medical degree:** Residents had a lower risk of recurrence than nonresidents, and M.D.s (Doctors of Medicine) had a lower risk than D.O.s (Doctors of Osteopathic Medicine).
278	Chauhan 2005[75]	Professional liability claims and Central Association of Obstetricians and Gynecologists members: Myth versus reality	The Kolmogorov-Smirnov test; the Student t test or Mann-Whitney test, paired t-test or Wilcoxon matched-pairs test	• **Gender:** Matched for years of practice, a case control comparison indicated that the litigation is significantly lower for female physicians
283	Walton 2020[51]	Profile of the most common complaints for five health professions in Australia	Poisson regression models	• **Gender:** Males were more than twice (120%) as likely to be the subject of a complaint than females ($P < 0.001$).<br>• **Age:** Age was also associated with risk of complaint; the risk was lowest for younger professionals (<35 years of age) and highest for those aged 45 years (3.1-fold higher in this group compared with the youngest group).<br>• **Place of birth:** Complaints were more common for overseas-born practitioners, unless from the UK or Ireland.<br>• **Geographic location:** There was some variation between jurisdictions within each profession, but on average Queensland tended to have slightly higher risk of complaints (24% more compared with NSW), whereas Victoria, South Australia and Western Australian tended to have a lower risk of complaints compared with NSW (19%, 22% and 33% lower respectively). Complaints for the Australian Capital Territory, Northern Territory and Tasmania were too few to comment.

284	Spittal 2015[83]	The PRONE score: an algorithm for predicting doctors' risks of formal patient complaints using routinely collected administrative data	Multivariate logistic regression	• **Gender, medical speciality, employment characteristics:** the PRONE (Predicted Risk Of New Event) score is a 22-point scoring system that indicates a doctor’s future complaint risk based on four variables: a doctor’s specialty and sex, the number of previous complaints and the time since the last complaint.
285	Carlson 2018[52]	Provider and practice factors associated with emergency physicians' being named in a malpractice claim	Logistic regression model	• **Practice experience, workload:** Increasing total number of years in practice (adjusted odds ratio 1.04; 95% confidence interval 1.02 to 1.06) and higher visit volume (adjusted odds ratio 1.09 per 1,000 visits; 95% confidence interval 1.05 to 1.12) were associated with being named in a malpractice claim.
289	Schaffer 2021[53]	Rates and characteristics of medical malpractice claims against hospitalists	Poisson rates and compared using a Z-test.	• **Medical speciality:** Compared with emergency medicine physicians, with whom hospitalists are sometimes compared due to both specialties being defined by their site of practice and the absence of longitudinal patient relationships, hospitalists had a significantly lower claims rate (1.95 vs 4.07 claims per 100 physician-years; P < .001).
306	Yates 2010[87]	Risk factors at medical school for subsequent professional misconduct: multicentre retrospective case-control study	Univariate and multivariable conditional logistic regression	• **Gender, social class, previous behaviour at school:** Male sex, lower estimated social class, difficulties in early/preclinical course, and delayed graduation were associated with case status. There was a less significant association with poorer progress in the clinical course. Multivariable analysis showed that male sex (odds ratio 9.80, 95% confidence interval 2.43 to 39.44, P=0.001), lower social class (4.28, 1.52 to 12.09, P=0.006), and failure of early or preclinical examinations (5.47, 2.17 to 13.79, P<0.001) were independently associated with being a case.
310	Samenow 2012[35]	The role of family of origin in physicians referred to a CME course	Significant test	• **Gender, ethnicity, marital status, medical speciality, teamwork, type of facility:** Physicians referred for sexual misconduct were predominantly male, white, married, middleaged, from solo or group practices (versus hospital practices) and represented a variety of medical subspecialties. The comparison group included more female physicians (p < 0.0001), more medical (p < 0.0001) and surgical (p <0.01) specialties, fewer generalists (p < 0.0001), fewer divorced physicians (p < 0.0001) and more physicians who indicated an academic/university practice (p < 0.0001). The sexual misconduct group had more ethnic diversity represented than the comparison group.<br>• **Family background:** The number of physicians in the disengaged and rigid group represents the largest subset of the physicians accused of sexual misconduct (35% of total cohort) completing the FACES II questionnaire. There were significantly more physicians in this pattern than the 15 other patterns (p＜0.05), even after adjusting for multiple comparisons.
313	Unwin 2015[94]	Sex differences in medico-legal action against doctors: a systematic review and meta-analysis	A random effect meta-analysis model	• **Gender:** Male doctors were significantly more likely to have experienced a medico-legal action in all three strata (disciplinary action: OR, 2.95; 95 % CI, 2.12–4.10; malpractice: OR, 1.74; 95 % CI, 1.11–2.71; other: OR, 2.46; 95 % CI, 2.05–2.94). There was considerable heterogeneity present in the strata disciplinary action (I2, 93.2 %) and malpractice experience (I2, 92.2 %).
320	Ambady 2002[86]	Surgeons' tone of voice: A clue to malpractice history	Logistic regressions	• **Interaction with patients:** Controlling for the full and brief vocal content variables, surgeons who were judged to be more dominant (OR 2.74, P = .02, 95% CI 1.16 to

				6.43) and less concerned/anxious on the basis of their tone of voice (OR 0.46, P = .05, 95% CI 0.21 to 1.01) were more likely to have been sued than surgeons who were judged to be less dominant and more concerned/anxious.
323	Mehtsun 2013[36]	Surgical never events in the United States	Multivariable logistic regression	• **Employment characteristics:** Physicians with clinical privilege or state licensure disciplinary action reports had an increased adjusted odds ratio(aOR = 1.73; 95% CI, 1.47–2.03) of being named in multiple surgical never event claims compared to physicians who did not have clinical privilege or state licensure disciplinary action reports.
326	Austin 2021[10]	Systematic review of the factors and the key indicators that identify doctors at risk of complaints, malpractice claims or impaired performance	Systematic review	• Twenty-three key factors identified, which were categorised as demographic or workplace related. Gender, age, years spent in practice and greater number of patient lists were associated with higher risk of malpractice claim or complaint. Risk factors associated with physician impaired performance included substance abuse and burn-out.
335	Papadakis 2004[77]	Unprofessional behavior in medical school is associated with subsequent disciplinary action by a state medical board	Independent t test	• **Previous behaviour at school:** There was a small, but statistically significant, difference in undergraduate GPA (3.3 for the case group and 3.4 for the control group; p = .04). Logistic regression analysis showed that disciplined physicians were more likely to have Concern/Problem/Extreme excerpts in their medical school file (odds ratio, 2.15; 95% confidence interval, 1.15–4.02; p = .02).
336	Nassiri 2019[54]	Unsolicited patient complaints among otolaryngologists	Wilcoxon rank sum test, Kruskal-Wallis rank sum test, Chi-square test	• **Medical speciality:** Otolaryngologists had a significantly greater average number of complaints per physician (6.4 UPCs) compared to all nonsurgical specialties (3.5 UPCs) and all other surgical specialties combined (5.3 UPCs; P ＜.001).<br>• **Gender, location of education:** A multivariate regression evaluating demographic factors including sex, medical school graduation location, subspecialty, board certification status, and practice setting revealed that male otolaryngologists (P = .0070) and those who graduated from a US medical school (P = .0036) were more likely to have a greater number of UPCs.<br>• **Complaint types:** An analysis of complaint types within the ‘‘high-risk’’ group of physicians, defined as the 95th percentile for number of complaints, demonstrates that dissatisfaction with treatment was again most common (38.1%), followed by dissatisfaction with accessibility (21.3%), communication (18.8%), patient concern (14.2%), and billing (7.7%) (P＜.001, ANOVA)
337	Raldow 2021[55]	Unsolicited patient complaints among radiation, medical, and surgical oncologists	Kruskal-Wallis test	• **Gender, medical speciality, graduation time, type of facility, geographic location:** The mean number of UPCs per physician was significantly associated with sex, practice setting, geographic region of practice, and oncologic specialty (P < .05). Surgical oncologists had significantly higher numbers of UPCs and radiation oncologists had significantly lower numbers of UPCs than medical oncologists. A more recent medical school graduation year and an academic practice setting were also associated with higher UPCs.
338	Kohanim 2016[76]	Unsolicited patient complaints in ophthalmology	Manne Whitney U test, chi-square tests	• **Medical speciality:** Ophthalmologists had significantly fewer complaints per physician than other nonophthalmic surgeons and nonsurgeons in the database (P < 0.001).

				• **Type of facility, gender, age:** Ophthalmologists from academic centers, female ophthalmologists, and younger ophthalmologists had significantly more complaints (P < 0.01), and general ophthalmologists had significantly fewer complaints than subspecialists (P < 0.05). After adjusting for covariates using multivariable analysis, working at an academic center was a statistically significant risk factor (adjusted relative risk, 1.82; 95% confidence interval, 1.36e2.43; P < 0.001).
341	Boyll 2017[78]	Variables that impact medical malpractice claims involving plastic surgeons in the United States	Univariate logistic regression, multiple logistic regression	• **Practice experience, interaction with patients:** According to the univariate logistic regression, years in practice, inform patient about possible consequences if he or she refuses treatment, provide patients with procedure-specific educational brochures had significant lower odds ratios. The use of procedure-specific brochures was associated with a significant reduction in the likelihood of being sued (OR [95% CI] = 0.02 [0.002, 0.29]; P = 0.004)<br>• **Continuous education:** Respondents whose insurance providers required periodic educational courses were less likely to be sued (OR [95% CI] = 0.40 [0.17, 0.96]; P = 0.04).
343	Adamson 2000[79]	The virtuous orthopaedist has fewer malpractice suits	T tests and Pearson correlations	• **Interaction with patients:** Physicians with better rapport with their patients, who took more time to explain, and who were available had fewer malpractice suits.
347	Tsimtsiou 2014[92]	What is the profile of patients thinking of litigation? Results from the hospitalized and outpatients' profile and expectations study	Wald-type F test.	• **Practice setting:** Outpatient status (2.4%, p=0.02) were associated with higher possibility to consider suing their physician (7.6%, p=0.002).
349	Gogos 2011[37]	When informed consent goes poorly: a descriptive study of medical negligence claims and patient complaints	χ2 tests.	• **Medical speciality:** The rate of complaints against plastic surgeons was significantly higher than that against any other type of specialist.
353	Tibble 2018[56]	Why do surgeons receive more complaints than their physician peers?	Multivariate model	• **Age:** Compared to surgeons aged 35 years or younger, surgeons aged 65 years and older were at the highest risk of complaints.(IRR = 6.57, 95% CI = 4.61–9.37).<br>• **Gender:** After adjusting for age, surgical subspecialty, location and clinical hours worked, male surgeons were 1.31 (95% CI = 1.09–1.57) times more likely to be the subject of a complaint compared with their female peers.<br>• **Geographic location:** Those who practiced in regional and rural areas were more likely to be the subject of a complaint compared with their metropolitan peers(IRR = 1.22, 95% CI = 1.07–1.39).<br>• **Medical speciality:** Surgeons who specialised in neurosurgery, plastic surgery and orthopaedic surgery were at higher risk of complaint compared with general surgeons (IRR = 1.75, 1.74 and 1.30, respectively).
